# Host Plant and Antibiotic Effects on Scent Bouquet Composition of *Anastrepha ludens* and *Anastrepha obliqua* Calling Males, Two Polyphagous Tephritid Pests

**DOI:** 10.3390/insects11050309

**Published:** 2020-05-14

**Authors:** Martín Aluja, Gabriela Cabagne, Alma Altúzar-Molina, Carlos Pascacio-Villafán, Erick Enciso, Larissa Guillén

**Affiliations:** Instituto de Ecología, A.C.-INECOL, Red de Manejo Biorracional de Plagas y Vectores, Clúster Científico y Tecnológico BioMimic^®^, Carretera antigua a Coatepec 351, 91073 Xalapa, Veracruz, Mexico; gabriela.cabagne@inecol.mx (G.C.); alma.altuzar@inecol.mx (A.A.-M.); carlos.pascacio@inecol.mx (C.P.-V.); erick.enciso@inecol.mx (E.E.); larissa.guillen@inecol.mx (L.G.)

**Keywords:** sex pheromones, host plant, microbiota, Tephritidae, *Anastrepha*, speciation, Sterile Insect Technique, phenotypic plasticity, sequestered compounds

## Abstract

In insects, the quality of sex pheromones plays a critical role in mating success and can be determined by the ability of larvae/adults to accrue chemical precursors. We tested the host-quality-effect hypothesis by analyzing the chemical composition of scent bouquets emitted by calling males of two polyphagous tephritid species (*Anastrepha* *ludens* and *A*. *obliqua*) that originated from 13 fruit species representing diverse plant families. In *A*. *ludens*, we worked with an ancestral host (Rutaceae), nine exotic ones (Rutaceae, Anacardiaceae, Rosaceae, Solanaceae, Lythraceae), and two species never attacked in nature but that represent candidates for host-range expansion (Solanaceae, Myrtaceae). In *A*. *obliqua*, we tested an ancestral, a native, and an exotic host (Anacardiaceae), one occasional (Myrtaceae), and one fruit never attacked in nature (Solanaceae). We identified a core scent bouquet and significant variation in the bouquet’s composition depending on the fruit the larvae developed in. We also tested the possible microbial role on the scent bouquet by treating adults with antibiotics, finding a significant effect on quantity but not composition. We dwell on plasticity to partially explain our results and discuss the influence hosts could have on male competitiveness driven by variations in scent bouquet composition and how this could impact insect sterile technique programs.

## 1. Introduction

In insects, mating success is often based on the ability to attract the opposite sex through the production of sex pheromones. These consist of chemical compounds that are mainly synthesized *de novo* or based on metabolites sequestered from host plants [1,2,3] and, in some cases, by means of symbiotic microorganisms [4]. Ever since the pioneering studies by Butenandt et al. [5] on *Bombix mori* L. (Lepidoptera: Bombycidae), Berger [6] on *Trichoplusia ni* Hübner (Lepidoptera: Noctuidae), Silverstein et al. [7] on *Ips confusus* (LeConte) (Coleoptera: Curculionidae), and the seminal work on pheromone evolution in moths by Roelofs and Brown [8], thousands of publications have been produced and summarized in several review articles and book chapters [9,10,11,12].

Because they are so critical for mating, the question of whether pheromones should largely remain invariant and not be affected by larval or adult diet has been debated for years, largely based on work on female-produced pheromones of Lepidoptera [9,13,14,15]. In these cases, pheromone components are largely biosynthesized *de novo* via fatty acid synthesis from precursor acetyl CoA derived from common dietary carbohydrates or fat [10,16,17]. In some species, precursors must be derived during larval feeding [18,19], whereas in species in which adults feed on plant nectar (carbohydrates), some pheromone precursors can also be derived from adult feeding [20,21,22]. Actual composition (“blend”) of the pheromone does not appear to be affected by either larval or adult diet, although the amount of pheromone produced and released by a female can be affected [23,24]. However, insects from other taxa use different compounds as sex pheromones, produced by other biochemical pathways, and the composition (blend) of these may be influenced by larval or adult diet [9].

Adults of some species of tephritid flies exhibiting lek mating systems (calling arenas visited by females to select mating partners), such as *Bactrocera* spp., are strongly attracted to plants containing methyl eugenol [25,26,27], raspberry ketone [28], or zingerone [29]. In the case of methyl eugenol (ME), after consumption by adults, and depending on the *Bactrocera* species, it can be metabolized to 2-ally-4,5-dimethoxyphenol, (*E*)-coniferyl alcohol, (*Z*)-coniferyl alcohol, or (*Z*)-3,4-dimethoxycinnamyl alcohol. These compounds are stored in the rectal pheromonal glands and posteriorly used as components or precursors of the sex pheromone [29,30,31,32,33,34]. In more general terms, the quality (i.e., completeness of the compound mixture, ratios of the chemical elements, quantity of the released pheromone) of the sex pheromone depends, in many species of tephritids, on the adult diet quality. It has been shown that a poor-quality diet can influence pheromone composition and male mating success, even in cases when the suboptimal food was consumed by calling males the day prior to pheromone collection [3,35,36,37,38,39,40,41].

Whereas the effects of the larval diet on mating behavior are fairly well studied in Lepidoptera [9,18,24], in the case of tephritid male flies, it remains largely unexplored [42,43]. Therefore, here we examined the influence of the fruit species in which larvae developed on the effluvia released by calling sexually-mature *Anastrepha ludens* and *A. obliqua* males, two polyphagous species that use different quality hosts [44]. *Anastrepha ludens* can infest the fruit of ca. 38 plants spanning all the way from the ancestral hosts *Casimiroa edulis* La Llave (locally known as “White Sapote”) and *C*. *greggii* (Watson) Chiang (both Rutaceae), to the occasional host *Capsicum pubescens* Ruiz & Pav cv. Manzano [45,46], and the conditional host *Solanum lycopersicum* (Mill.) (both Solanaceae). *Anastrepha obliqua* is more specialized, preferring fruit within the Anacardiaceae (e.g., *Spondias mombin* L., S. *purpurea* L., *S*. *radlkoferi* Donn. Sm. and *M. indica*) but is also considered polyphagous since it has been reported infesting the fruit of *Psidium guajava* L. and *Myrciaria floribunda* (H. West ex Willd.) Berg (both Myrtaceae) [47,48], and *Averrhoa carambola* L. (Oxalidaceae) [49]. *Anastrepha ludens* is distributed from S. Texas to Costa Rica [50], while *A*. *obliqua* is distributed from N. Mexico to Argentina [46].

Motivated by a recent study documenting the fitness costs of polyphagy in *A. ludens* [44], here we tested the host-quality effect hypothesis using male scent bouquets as our experimental variable. The main sex pheromone components of *A. ludens* males include (*Z*)-3-nonen-1-ol, (*Z,Z*)-3,6-nonadien-1-ol, (*E*)-α-bergamotene, (*E,E*)-α-farnesene, (*E,Z*)-α-farnesene, suspensolide, anastrephin, and epianastrephin [51,52,53,54,55,56,57]. Of these compounds, the first five are shared by *A. ludens* and *A. obliqua*. Based on the results of Birke et al. [58] and Birke and Aluja [44], we predicted that the chemical composition of the scent bouquet collected from the effluvia released by sexually-mature calling *A. ludens* and *A. obliqua* males would differ depending on the host fruit in which larvae developed, and that this effect would be stronger in flies originating from non-natural hosts artificially infested under laboratory or field conditions (“conditional hosts”) [59]. Because it has recently been demonstrated that bacteria in the adult gut affect the sexual performance of tephritids [60,61,62], we also tested the effect of antibiotics on the chemical composition of the *A. ludens* scent bouquet. This work has important potential practical applications for tephritid fly area-wide management programs that apply the sterile insect technique (SIT), as the success of this technique relies on the ability of sterile male flies to copulate as many times as possible with wild females [63], which is influenced, among many other factors, by the quality of the sex pheromone of adult flies [35,36,37].

## 2. Materials and Methods

The *A. ludens* and *A. obliqua* male flies used in this study originated from either naturally field-infested fruit, fruit from forced field infestations with wild flies, or fruit from forced laboratory infestation with semi-wild laboratory flies. With the exception of *S. lycopersicum,* never reported as a host for both fly species, and *P. guajava,* never reported as host for *A. ludens,* all the remaining fruit tested have been recorded as natural or non-natural hosts *sensu* Aluja and Mangan [59] (Table 1). Details of field collection sites and forced infestation sites are presented in Table S1.

*Collection of male flies stemming from naturally infested fruit.* Infested fruit were collected in the field (details in Table S1), transported to the laboratory, and placed in 25 × 30 × 15 cm plastic baskets over a plastic tray with sterilized vermiculite as pupation medium on the bottom. Three days after collection, baskets were checked every three days to collect pupae. Recovered pupae were placed in 250 mL plastic containers with vermiculite. Containers were covered with a piece of pantyhose to allow for proper aeration. Pupae were moistened every third day until adult emergence which started to occur ca. 15 days after pupation. One day before fly emergence, containers with pupae were placed inside of 20 × 20 × 20 cm plexiglass cages with food (3:1 mixture of sugar: hydrolyzed protein) and water. Shortly after adult emergence, males and females were separated, placed in plexiglass cages and fed *ad libitum* with water and food in a laboratory at 26 ± 1 °C, 70 ± 5% RH, and 12:12 h L:D photoperiod.

*Forced infestations under field conditions*. The forced infestations with *A. ludens* on *S. lycopersicum* cv. ‘Saladette’, *P. persica* cv. ‘Criollo’, *P. guajava* cv. ‘Criolla’, and *C. pubescens* were performed based on methods described in Aluja et al. [64] using adult flies obtained from naturally infested *C.* × *paradisi* cv. ‘Ruby Red’. The infestation of *M.* × *domestica* cv. ‘Rayada’ and *M.* × *domestica* cv. ‘Golden Delicious’ was performed with wild flies stemming from naturally infested in *C. edulis.* For forced infestations with *A. obliqua,* we only used adult flies from *M. indica* cv. ‘Manila’. These flies were maintained in separate plexiglass cages of 20 × 20 × 20 cm with food (a 3:1 mixture of sugar: hydrolyzed protein) and water *ad libitum* for 12–15 days until sexually mature. Two weeks before the beginning of forced infestations in the field, we selected between one and 12 trees/bushes per plant species. From each individual plant/tree, 1–16 branches with 2–10 unripe fruit each (depending on size of fruit) were bagged with white chiffon bags to prevent damage to fruit by local herbivores. Two 12- to 15-day old *A. ludens* or *A. obliqua* gravid females per fruit were released into each bagged branch with fruit (only in the case of *A. obliqua* infesting *S. purpurea* did we use one female per fruit given the small fruit size). Flies were left inside bags for 72 h and then removed. In the case of *M*. x *domestica*, in which larval development is slower [65], fruit were left bagged for up to 15 additional days. Then, fruit were harvested and processed as described in the previous section.

*Forced infestations under laboratory conditions.* Only mango cv. ‘Manila’ was infested in the laboratory. The mangos were purchased in the supermarket as unripe fruit (prior to color break). Four to five mangos were thoroughly washed with tap water and placed in cages of 30 × 30 × 30 cm with 30 *A. ludens* females and 20 males. After 48 h, all fruit were removed from the cages and processed as described in the previous section.

*Antibiotics treatment*. Groups of 50 virgin males of *A. ludens* from field infested *P. persica* and from a laboratory colony reared on an artificial diet were kept in plexiglass cages of 30 × 30 × 30 cm at 26 ± 1 °C, 70 ± 5% RH, and 12:12 L:D photoperiod. This *A. ludens* laboratory colony has been maintained on an artificial diet at the Red de Manejo Biorracional de Plagas y Vectores of the Instituto de Ecología, A.C., Xalapa, Mexico (RMBPV) for over 120 generations and the last introduction of wild flies originating from citrus fruit to the colony was made four years prior to the study [66]. The larval artificial diet consisted of the following ingredients slightly modified from [67]: dried yeast (7.93% w/w), sugar (7.93% w/w), wheat germ (7.93% w/w), corncob fractions (11.9% w/w), citric acid (0.4% w/w), sodium benzoate (0.47% w/w), and water (63.44% w/w). Adult flies of this colony were maintained in plexiglass cages at a density of 0.069 adult flies per cm^3^. For the experiments, newly emerged flies were provided food *ad libitum* (a 3:1 mixture of sugar: hydrolyzed protein) and sterile water containing the antibiotics streptomycin (400 μg/mL) and rifampicin (400 μg/mL). Water was offered to flies in a sterile cotton ball saturated with 2 mL of the antibiotic’s solution in a plastic dish. Cotton balls were replaced daily for 15 days to guarantee that flies were ingesting a fresh solution of antibiotics. Six groups of 15 sexually-mature flies each were used for volatile collections as described in the following section. Flies reared identically but not exposed to antibiotics were used as controls. To confirm that bacteria were indeed affected after treatment with antibiotics, guts of control and antibiotics-treated males were dissected in sterile conditions and macerated in 100 μL of Luria–Bertani (LB) media. From this solution, 10 μL were plated on solid LB media and bacterial growth was observed after incubation during 24 h at 30 °C.

*Male effluvia collections.* Effluvia emanating from abdominal pouches and the proctiger of sexually-mature virgin calling *A. ludens* (10–15 day old) and *A. obliqua* (8–13 day old) males that developed as larvae in each of the host fruit we tested (Table 1) were collected by a dynamic aeration technique. Fifteen calling male flies from each origin were placed inside a collection chamber (glass jar of 12.4 long by 8.4 cm diameter, with an adapted cap for air inlet and outlet). A flow of purified air (1 L/min) went through each collection chamber to carry male volatiles to a volatile collection trap (VCT-1/4-3-HSQ-P, ARS) with super Q (odor adsorbent material) for two hours during the courtship and mating time *sensu* Aluja et al. [47] (from 15:00 to 17:00 h in the case of *A. ludens* and from 08:00 to 10:00 h for *A. obliqua*). Volatile traps were connected to a vacuum pressure system with an air flow of 1 L/min. The collected volatile compounds were eluted with 400 μL of dichloromethane (HPLC grade, Sigma-Aldrich) and stored in 2 mL amber glass vials at -80 °C until they were chemically analyzed. There were six experimental replicates for each fly origin. In the case of *A. ludens* males stemming from *P. communis, P. granatum, M.* × *domestica* cv. ‘Rayada’, and *M.* × *domestica* cv. ‘Golden Delicious’, volatiles were collected from a single group of 15 males (10–25 days old) during six-consecutive days (i.e., six pseudoreplicates per fruit).

*Chemical analyses of volatile compounds.* Volatiles were analyzed using a gas chromatograph (GC-2010 Plus, Shimadzu) with a ZB-5Msi column (30 m × 0.25 mm × 0.25 μm), coupled to a mass spectrometer (QP-2010 Ultrasystem, Shimadzu) (GC-MS). One microliter of volatile samples was injected into the GC in splitless mode at 250 °C, programmed for compound separation at an initial oven temperature of 50 °C for five min, then a ramp of 15 °C/min up to 280 °C for five min. For mass analysis, the ion source temperature was 200 and 250 °C for the interface. A solvent delay of three min was used. Helium was used as a carrier gas at a constant flow rate of one mL/min. Mass spectra of compounds were compared with those registered in the National Institute of Standards and Technology (NIST) library version 2.0D, NIST/EPA/NIH (NIST05) and confirmed by authentic standards in the cases of (*Z*)-3-nonen-1-ol, (*Z,Z*)-3,6-nonadien-1-ol (purchased in Sigma Aldrich, Mexico), (*E,E*)-α-farnesene and (*Z*)-β-farnesene (purchased in Toronto Research Chemicals, TRC-Canada), and anastrephin and epianastrephin (donated by D. Kuzmich, USDA). Quantification was achieved using a calibration curve with six concentrations of the authentic commercial standard fitted to a linear regression with a coefficient of determination higher than 0.99.

*Statistical analyses.* We used a one-way analysis of variance (ANOVA) to test the null hypothesis that the mean concentration of the chemical compounds identified in the volatiles from adult flies was the same regardless of the host plant species in which fly larvae developed. The assumptions of homoscedasticity and normality were checked graphically with the model residuals. A Box-Cox analysis was used to examine the power of the transformation that would minimize residual variation and correct severe heteroscedasticity of the residuals of models fitted to untransformed data [68]. Based on this analysis, the following transformations were applied: in the tests with *A. ludens*, the concentrations of (*Z,Z*)-3,6-nonadien-1-ol, (*E,E*)-α-farnesene, and (*Z*)-3-nonen-1-ol were log transformed; the concentrations of epianastrephin and anastrephin were inverse sqrt transformed. In the tests with *A. obliqua*, the concentrations of (Z,Z)-3,6-nonadien-1-ol, (*E,E*)-α-farnesene, and (*Z*)-β-farnesene were inverse sqrt, log 10 and y^−1.69^, respectively, transformed. When significant effects were detected by ANOVA (*p* < 0.05), multiple comparisons of means were performed using Tukey contrasts. These analyses were run separately for *A. ludens* and *A. obliqua*. In the case of the tests with *A. ludens*, flies from *M. x domestica* cv. ‘Golden Delicious’, *M. x domestica* cv. ‘Rayada’, *P. communis*, and *P. granatum* were not considered in the analyses, as observations from these hosts were not based on true replicates. To obtain a better overview of the natural clustering patterns of scent-bouquet compound presence and concentration (peak area) as influenced by the different fruit tested, hierarchical clustering and heat map analyses were performed with the software MetaboAnalyst 4.0 [69], using normalized data, Euclidean distance measures, and the Ward clustering algorithm.

In the experiment with the antibiotic treatment, we used a two-sample t-test to test the null hypothesis that the mean values of the chemical compound concentrations in the scent bouquet of flies from the antibiotic treatment and the control group were equal.

ANOVA and t-tests were performed with the software R [70] using the package “Multcomp” [71], and the level of significance was set at *p* < 0.05. The Box-Cox analysis was performed with the Design-Expert^®^ 10 software (Stat-Ease, Inc, Minneapolis, MN).

## 3. Results

### 3.1. Host Plant Effects on Male Effluvia Composition

**Figure 1:**
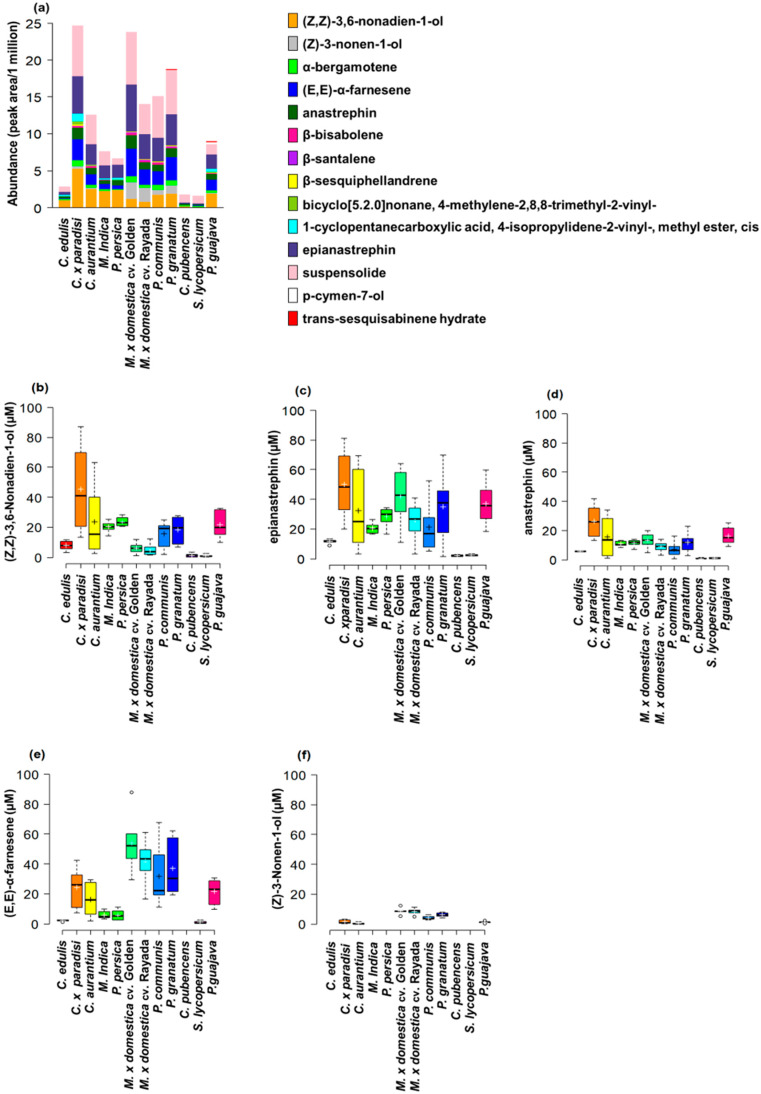
Chemical compounds identified in the effluvia (scent bouquets) of sexually-mature calling *A. ludens* males developed as larvae in different hosts. (**a**) The abundance (peak area/one million) of the 14 compounds tentatively identified by comparing mass spectra registered in the National Institute of Standards and Technology (NIST) library or confirmed with authentic standards. Note that the color bars of some compounds are extremely narrow and almost undetectable because such compounds were identified in relatively small abundances (the exact abundances of the compounds identified are available in Table S2). (**b**–**f**) Boxplots of the concentration of the compounds confirmed with authentic standards; the box shows the interquartile range and the horizontal line in each box the median, the whiskers indicate the minimum and maximum values, open circles indicate outliers, and the cross inside the box the mean. “Golden” used as abbreviation for ‘Golden Delicious’ in the case of *M.* × *domestica*.

***Anastrepha ludens.*** We identified 14 chemical compounds in the effluvia (scent bouquets) of sexually-mature calling *A. ludens* males through GC-MS (Figure 1a, Table S2). Of these, (*Z,Z*)-3,6-nonadien-1-ol, α-bergamotene, anastrephin, epianastrephin, and suspensolide are known sex pheromone components [53,55] that were present across flies from all the fruit tested as larval rearing media (Figure 1a, Table S2). Interestingly, the scent bouquet of flies originating from the ancestral host *C. edulis* was less concentrated and contained fewer compounds (seven) than the scent bouquet of flies originating from the commonly infested exotic host *C*. x *paradisi*, also Rutaceae (12 compounds), or the non-host *P. guajava*, Myrtaceae (12 compounds) (Figure 1a, Table S2).

We note that the scent bouquet of *A*. *ludens* flies originating from *C*. x *paradisi* and *P*. *guajava* had five additional chemical compounds; *P*. *communis* and *P*. *granatum* had four; *C*. *aurantium* and *M*. x *domestica* (cv. ‘Rayada’ and ‘Golden Delicious’) had three; and *M*. *indica* and *P. persica* had one additional chemical compound compared to the scent bouquet of flies from the ancestral host *C. edulis.* In contrast, males originating from *S. lycopersicum* and *C. pubescenses* (both Solanaceae) produced a scent bouquet containing one and two, respectively, fewer compounds than that of *C*. *edulis* (Table S2). Among the compounds identified in the scent bouquet of *A. ludens*, p-cymen-7-ol was unique to the flies developed in *P. guajava,* and trans-sesquisabinene was only detected in flies from *P. guajava* and *P. granatum* (Figure 1a, Table S2).

Of the chemical compounds that we confirmed with authentic standards, (*Z,Z*)-3,6-nonadien-1-ol, epianastrephin, and anastrephin were found in the scent bouquets of flies from all the host fruit considered in the study. (*E,E*)-α-farnesene was not detected in flies from *C. pubescens*, and (*Z*)-3-nonen-1-ol was detected in flies from *C. aurantium*, *C. x paradisi*, *M. x domestica* cv. ‘Golden Delicious’, *M. x domestica* cv. ‘Rayada’, *P. communis*, *P. granatum,* and *P. guajava* (Figure 1b–f).

The concentration of (*Z,Z*)-3,6-nonadien-1-ol varied significantly as a function of the host plant species in which larvae developed (F_7, 40_ = 23.38, *p* < 0.0001). On average, the scent bouquets of flies from *C. pubescens* and *S. lycopersicum* had the lowest concentrations of (*Z,Z*)-3,6-nonadien-1-ol, whereas the ones in flies from *C. x paradisi* had the highest (Figure 1b; Supplementary File S1). The same trend was observed with the concentration of epianastrephin, which was significantly higher in the scent bouquet of flies from *C. x paradisi*, whereas the lowest concentrations were observed in flies from *C. pubescens* and *S. lycopersicum* (F_7, 40_ = 44.84, *p* < 0.0001; Figure 1c, Supplementary File S1). The concentration of anastrephin was significantly influenced by the host fruit in which flies developed (F_7, 40_ = 14.02, *p* < 0.0001) and the highest concentration was again observed in the scent bouquet of flies from *C. x paradisi*, followed by *P. guajava* and *C. aurantium*, whereas the lowest concentrations were observed in the scent bouquets of flies originating from *C. pubescens* and *S. lycopersicum* (Figure 1d; Supplementary File S1). The concentration of (*E,E*)-α-farnesene also varied significantly as a function of the host fruit in which larvae developed (F_6, 35_ = 19.08, *p* < 0.0001). We found the highest and similar concentrations of (*E,E*)-α-farnesene in the scent bouquets of flies from *P. guajava*, *C. aurantium*, and *C. x paradisi*, whereas the lowest concentrations were observed in the scent bouquets of flies from *S. lycopersicum* and *C. edulis* (Figure 1e; Supplementary File S1). Finally, the concentration of (*Z*)-3-nonen-1-ol did not differ significantly among the scent bouquets of flies originating from *P. guajava, C. aurantium*, and *C. x paradisi* (F_2, 15_ = 3.3, *p* = 0.0646; Figure 1f).

**Figure 2:**
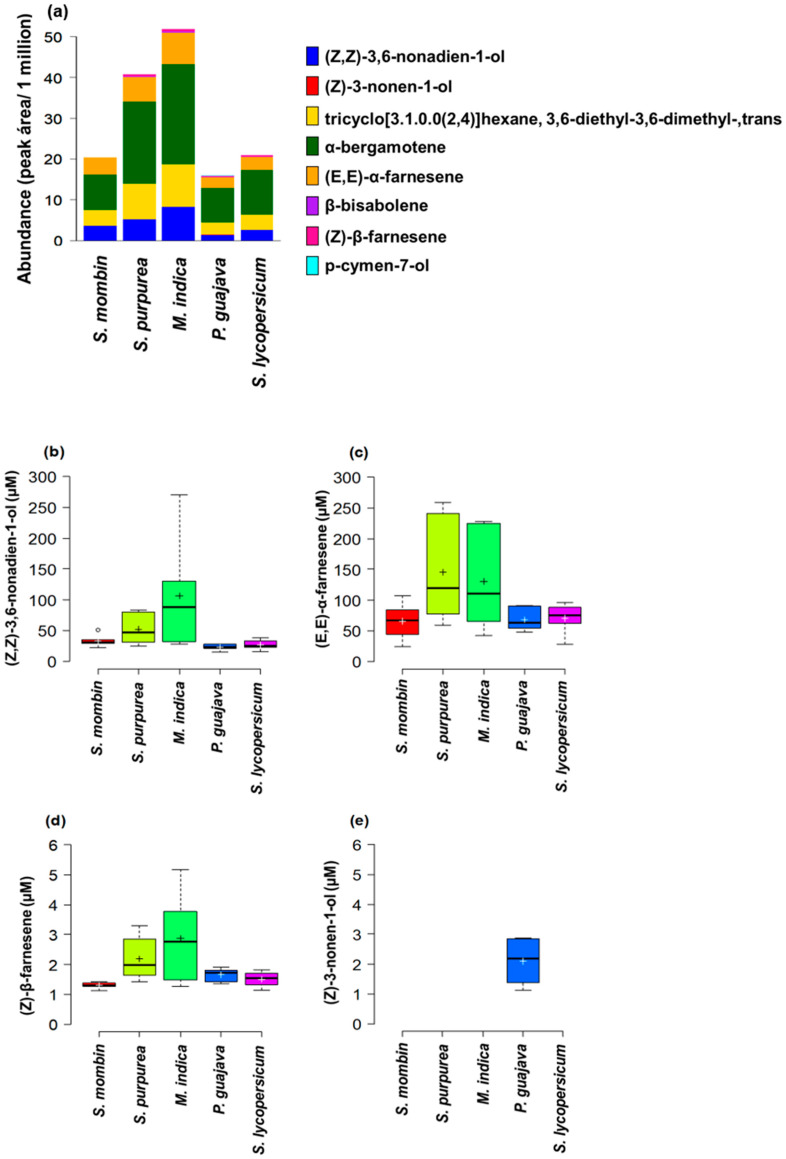
Chemical compounds identified in the effluvia (scent bouquets) of sexually-mature calling *A. obliqua* males developed as larvae in different fruit. (**a**) The absolute abundance (peak area/one million) of the eight compounds tentatively identified by comparing mass spectra registered in the NIST library or confirmed with authentic standards. Note that the color bars of some compounds are extremely narrow and almost undetectable because such compounds were identified in very small abundances (the exact abundances of the eight compounds are available in Table S2). (**b**–**e**) Boxplots of the concentration of the compounds confirmed with authentic standards; the box shows the interquartile range and the horizontal line in each box the median, the whiskers indicate the minimum and maximum values, open circles indicate outliers, and the cross inside the box the mean.

***Anastrepha obliqua.*** We identified up to eight chemical compounds in the scent bouquet of *A. obliqua* through GC-MS (Figure 2a, Table S2). Only the scent bouquet of flies from *P. guajava*, a rare natural host in Mexico found infested in few localities by *A*. *obliqua*, contained the eight compounds, whereas the scent bouquet of flies from the ancestral host *S. mombin* contained only four compounds. We noted that the scent bouquet of sexually-mature calling *A. obliqua* males originating from *P. guajava, S. purpurea*, *S. lycopersicum*, and *M. indica*, had four, two, two, and two additional chemical compounds, respectively, than the scent bouquet of flies from the ancestral host *S. mombin* (Table S2). The compounds (*Z*)-3-nonen-1-ol and p-cymen-7-ol were only identified in very low concentrations in the scent bouquet of flies from *P. guajava* (Figure 2a, Table S2).

Of the chemical compounds identified in the scent bouquet of *A. obliqua* males confirmed with authentic standards, the compounds (*Z,Z*)-3,6-nonadien-1-ol, (*E,E*)-α-farnesene, and (*Z*)-β-farnesene were found in the scent bouquet of flies from all the host fruit considered in the study, whereas (*Z*)-3-nonen-1-ol was only detected in flies originating from *P. guajava* (Figure 2b–e).

The concentration of (*Z,Z*)-3,6-nonadien-1-ol in the scent bouquet of *A. obliqua* males varied significantly as a function of the host fruit in which larvae developed (F_4, 24_ = 5.5, *p* = 0.0026). On average, the highest concentration was observed in the scent bouquet of flies from *M. indica*, but this concentration was only significantly different from the concentration observed in the scent bouquet of flies originating from *P. guajava* and *S. lycopersicum* (Figure 2b, Supplementary File S1). Although the scent bouquet of flies originating from *M. indica* and *S. purpurea* had the highest concentration of (*E,E*)-α-farnesene (Figure 2c), we found no statistically significant effect of the host fruit in which fly larvae developed in the concentration of this compound in the scent bouquet of *A. obliqua* (F_4, 25_ = 2.45, *p* = 0.0726). The concentration of (*Z*)-β-farnesene differed significantly among flies from the different host fruit (F_4, 24_ = 4.66, *p* = 0.0063); on average, the scent bouquet of flies originating from *M. indica* and *S. purpurea* had the highest concentrations of this compound, but these concentrations were only significantly different from that observed in flies stemming from *S. mombin* (Figure 2d, Supplementary File S1).

**Figure 3:**
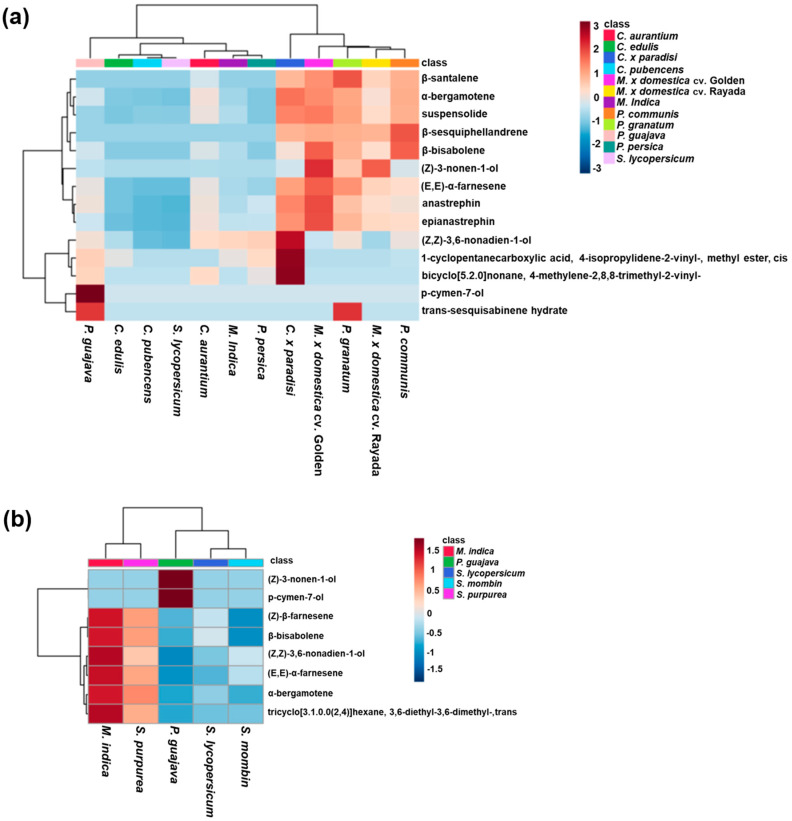
Heatmaps showing the hierarchical clustering of mean abundances in chemical compounds (peak area) related with the volatiles contained in the effluvia (i.e., scent bouquet) released by sexually-mature calling males of two species within the highly derived *fraterculus* species group: (**a**) *A. ludens* and (**b**) *A. obliqua.* Note that the scent bouquet of *P. guajava* (conditional host) and *C. x paradisi* (preferred host) are the most contrasting in *A. ludens*, and, in the case of *A*. *obliqua* again, *P. guajava*, an occasional host, is the most contrasting. “Golden” used as abbreviation for ‘Golden Delicious’ in the case of *M. x domestica.*

The hierarchical clustering, based on all chemical compounds and their mean abundance (i.e., peak area) identified in the effluvia of sexually-mature calling *A*. *ludens* males originating from different fruit species, indicates that the scent bouquet profiles of the occasionally used hosts *M. x domestica*, *P. communis* (both Rosaceae), and *P. granatum* (Lythraceae) exhibit strong similarities (second group in the primary separation shown by the dendrogram on top of Figure 3a). Interestingly, this group is closer to the scent bouquet profile of males stemming from *C. x paradisi* (Rutaceae)*,* one of the most-preferred hosts of *A. ludens*. The volatile profiles of males from these hosts have higher abundances in nine shared-chemical compounds (first group in the primary separation of the lateral dendrogram in Figure 3a). The only difference of this group with respect to *C. x paradisi* is related to (*Z,Z*)-3,6-nonadien-1-ol, cyclopentanecarboxylic acid, 4-isopropylidene-2-vinyl-, methyl ester, cis and bicyclo [5.2.0] nonane, 4-methylene-2,8,8-trimethyl-2-vinyl-, which were more abundant in this preferred natural host. This analysis also flushed out the fact that the scent bouquet of flies originating from guava was more dissimilar to the rest of the treatments with higher abundances of p-cymen-7-ol and trans-sesquisabinene (Figure 3a). In the case of *A. obliqua,* the hierarchical clustering analysis shows that *M. indica* and *S. purpurea* are the most similar between them and, repeating the phenomenon observed in *A*. *ludens*, that the profile from *P. guajava* is the most contrasting, exhibiting the highest concentrations of (*Z*)-3-nonen-1-ol and p-cymen-7-ol (Figure 3b).

### 3.2. Antibiotic Treatment Experiment

**Figure 4:**
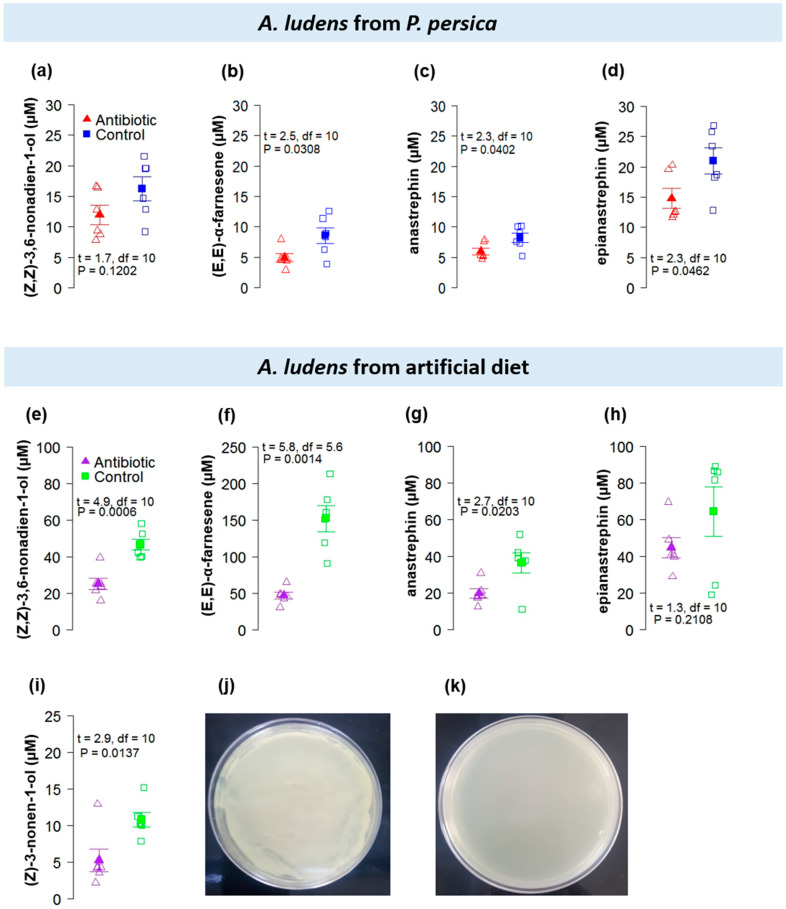
The concentration of the chemical compounds confirmed with authentic standards collected in the effluvia of sexually-mature calling *A. ludens* males from (**a**–**d**) *Prunus persica* and (**e**–**i**) from artificial diet after antibiotic treatment administration for 12–15 days to newly emerged adult flies. Solid symbols indicate mean values (±SE) and open symbols indicate jittered data points. (**j**,**k**) Petri dishes depicting the bacterial culture of guts of *A. ludens* in Luria–Bertani (LB) medium: Untreated control (**j**) and treatment with the combination of the antibiotics streptomycin and rifampicin (**k**); note the profuse bacterial growth in (**j**) and lack of bacterial growth in the LB medium plated with gut samples stemming from flies treated with antibiotics (**k**).

Overall, the effluvia from sexually-mature calling males originating from larvae reared in *P. persica* (Figure 4a–d) or a laboratory diet (Figure 4e–i), contained lower concentrations of known sex pheromone components when the adults were treated with antibiotics than when they were free of them. With respect to the components of the scent blend, the one collected from males originating from *P*. *persica* contained seven chemical compounds including (*Z,Z*)-3,6-nonadien-1-ol, α–bergamotene, 1-cyclopentanecarboxylic acid, 4-isopropylidene-2-vinyl-, methyl ester, cis, (*E,E*)-α –farnesene, suspensolide, anastrephin, and epianastrephin (Table S2) and the one from males originating from the artificial diet contained 12 compounds including (*Z*)-3-nonen-1-ol, (*Z,Z*)-3,6-nonadien-1-ol, α-bergamotene, β-santalene, 1-cyclopentanecarboxylic acid, 4-isopropylidene-2-vinyl-, methyl ester, cis, bicycle [5.2.0] nonane, 4-methylene-2,8,8-trimethyl-2-vinyl-, (*E,E*)-α-farnesene, suspensolide, β-bisabolene, tricyclo[3.1.0.0(2,4)]hexane,3,6-diethyl-3,6-dimethyl-, trans-, anastrephin, and epianastrephin (Table S3). Notably, flies originating from the artificial diet produced higher amounts of effluvia compared to those stemming from *P. persica.* Bacterial growth in LB medium was observed in gut samples from control flies but not from males treated with antibiotics (Figure 4j,k).

## 4. Discussion

We found a strong influence of the fruit in which *A. ludens* and *A*. *obliqua* larvae were reared on the composition (i.e., number of blend components and quantity thereof) of the scent bouquet detected in the effluvia of sexually-mature calling males. If indeed this finding is the result of a carry-over effect of sequestered compounds by the larvae to the adults that then use these precursors to synthetize the novel compounds in the odor bouquet, or if the phenomenon of additional compounds showing up in the odor bouquet released in the effluvia of calling males is a result of environmentally-driven (i.e., chemical environment in which the larvae developed) phenotypic plasticity, this requires additional research. We also found that treating males with antibiotics (mixture of streptomycin and rifampicin) did not modify the scent bouquet composition, but did significantly reduce the quantity of known sex pheromone components (i.e., reported previously in the literature and confirmed here with authentic standards) released by calling males. Interestingly, we discovered that the scent bouquet of males originating from ancestral hosts (*C*. *edulis* and *S*. *mombin* in *A*. *ludens* and *A*. *obliqua*, respectively) contained the least number of components (proposed here as the “core” blend) and that males originating from the most “novel” hosts, produced the richest scent bouquets, some with compounds never reported before, such as β-santalene, cyclopentanecarboxylic acid, 4-isopropylidene-2-vinyl-, methyl ester, cis, β-sesquiphellandrene, bicyclo[5.2.0]nonane, 4-methylene-2,8,8-trimethyl-2-vinyl-, p-cymen-7-ol, and trans-sesquisabinene hydrate for *A. ludens* (Supplementary Table S2)*,* and tricyclo[3.1.0.0(2,4)]hexane, 3,6-diethyl-3,6-dimethyl-, trans and p-cymen-7-ol for *A. obliqua* (Supplementary Table S2),. At this stage, we cannot ascribe any behavioral role of these “novel compounds” as we still need to perform electrophysiological, wind tunnel, and field cage tests, but given that invariably all scent bouquets included known sex pheromone components, we can reasonably surmise that it is possible that the added components to the “core” blend could likely influence the response of females to males producing more complex scent bouquets. This we will test in future studies. But independent of the “attractiveness” of males originating from different fruit, there is evidence indicating that male mating success (e.g., total number of copulations per male) can indeed be influenced by the larval host. For example, in the case of *A. ludens*, males stemming from *C. x paradisi* called and mated significantly more than males stemming from *Casimiroa greggii* (Watson) (Sapindales: Rutaceae), one of the two ancestral hosts of this tephritid species [72]. In addition, in the case of *A*. *obliqua*, male larval diet influenced female fertilization success and copula duration [73]. These authors discovered an intriguing interaction between larval feeding substrate (host fruit) and male adult diet: Poorly fed males (deprived of protein) originating from the ancestral host *S*. *mombin* mated over significantly shorter periods compared to males originating from the exotic host *M*. *indica* [73]. Shelly [43] also documented that melon fly males, *Zeugodacus cucurbitae* (Coquillett), had a higher mating success when larvae were reared in zucchini than when reared in papaya.

The 14 scent bouquet components reported here for *A. ludens* males stemming from different fruits include (*Z*)-3-nonen-1-ol, (*Z,Z*)-3,6-nonadien-1-ol, α-bergamotene, (*E,E*)-α-farnesene, suspensolide, β-bisabolene, anastrephin, and epianastrephin (Figure 1; Supplementary Table S2), which have been reported previously as forming part of the sex pheromone of this fruit fly species [51,52,53,54,55,56,57] (Supplementary Table S4). Except for (*Z*)-3-nonen-1-ol, (*E,E*)-α-farnesene, and β-bisabolene, all these volatile compounds were detected in the effluvia of calling males originating from all fruit we tested as larval rearing media. In the case of *A. obliqua,* up to five sex pheromone compounds were found: (*Z,Z*)-3,6-nonadien-1-ol, α-bergamotene, (*E,E*)-α-farnesene, (*Z*)-β-farnesene, and β-bisabolene, all of them previously reported in this species [74,75,76,77,78,79]. Of the latter compounds, (*Z,Z*)-3,6-nonadien-1-ol, (*E,E)-*α-farnesene, and α-bergamotene were detected in calling *A. obliqua* males originating from all the different larval rearing media we chose to test the “host-quality-effect hypothesis” (Figure 1 and Figure 2; Supplementary Table S2). Importantly, in *A. ludens* we discovered stark differences in the scent-bouquet blend profiles and in the abundances of its components influenced by the larval diet (i.e., host fruit), with as many as seven compounds appearing when compared to the blend observed in males stemming from the ancestral host *C*. *edulis* (tentatively identified as (*Z*)-3-nonen-1-ol, β-sesquiphellandrene, β-santalene, β-bisabolene, bicyclo[5.2.0]nonane, 4-methylene-2,8,8-trimethyl-2-vinyl-, p-cymen-7-ol, trans-sesquisabinene hydrate). In the case of *A*. *obliqua*, the same phenomenon was observed but with fewer different compounds detected when comparing its ancestral host *S*. *mombin* with other ones (identified as (*Z*)-β-farnesene, β-bisabolene, (*Z*)-3-nonenol, and p-cymen-7-ol). Remarkably, the simplest scent bouquet was observed in adults stemming from both ancestral hosts (*C*. *edulis* and *S*. *mombin* for *A*. *ludens* and *A*. *obliqua*, respectively), which we treat here as the “core scent bouquet”, for discussion purposes, as well as in *A*. *ludens* adults originating from the two representatives of the Solanaceae (*C*. *pubescens* and *S*. *lycopersicum* cv. ‘Saladette’), an occasional, natural and a conditional, non-natural host. Why these two solanaceous plants had such a strong effect on the scent bouquet composition merits further investigation. Our findings resemble in part the ones by Merli et al. [42], who found that the volatile profile of the sex pheromone of *C. capitata* varied significantly as a function of the larval food even though these authors worked with many fewer host plants and did not report novel compounds.

Here we also discovered high variability in the abundances of certain scent bouquet components (Figure 1 and Figure 2). Consistent with the ancestral host/core scent bouquet concept we put forth, our study revealed that male flies from the ancestral hosts produced less amounts of the various scent bouquet components compared to males from more recently used natural hosts or artificially infested ones (Figure 1 and Figure 2). As noted before, the lowest amounts of scent bouquet compounds were observed in flies stemming from low-quality hosts [44], within the Solanaceae. Males from ancestral hosts produced a simple and low concentration scent bouquet which, in theory, could smell different from the more concentrated scent bouquet released by males reared on non-host fruit such as guava. Under such a scenario, the question arises as to what scent is more sexually attractive to female flies and under which circumstances? Addressing this question will improve our understanding about how larval food influences the ability of adult males to obtain sexual partners and perpetuate their genes to the next generation. In addition, this information could be useful in developing artificial larval diets to rear *A. ludens* and *A. obliqua* males with a scent bouquet highly attractive to wild females. This could improve the effectiveness of SIT-based programs against *A. ludens* and *A. obliqua* whose success relies on the ability of sterile male flies to mate and induce sterility in wild females [80].

The clear host plant effects on scent bouquet composition possibly suggests that larvae sequestered bouquet components or precursors that were carried over to the adult through the pupal stage (metamorphosis) (host-quality-effect hypothesis). How could the scent bouquet precursors apparently accrued by larvae in the host they fed on be carried over to the adults when there is an intermediate stage (i.e., pupae) in the metamorphosis phenomenon? Are novel odor bouquet components a cheap byproduct of the metabolization process of the secondary chemicals in the hosts or do they represent costly additional metabolic synthetic routes conveying competitive advantages to males? And which is the role of these novel compounds in the scent bouquet blend in adult behavior? All these questions require further investigation. Based on previous work [81], we surmise that the ingested/sequestered compounds by the larvae could be absorbed by fat cells that reach the adult stage unchanged when metamorphosis is complete. We plan to test this by marking a few precursors and testing if they indeed are released into the hemolymph of the adult once fat cell death finally takes place [81]. Alternatively, the phenomenon we observed could be also ascribed to plasticity, as recently Dion and collaborators [82] were able to document that the composition of the male sex pheromone (absolute quantities and ratios) of the butterfly *Bicyclus anynana* (Butler) (Nymphalidae) is influenced by the temperatures experienced by larvae and adults. These authors [82], citing West–Eberhard [83] and Forsman [84], defined phenotypic plasticity as “the ability of a genotype to produce different phenotypes in response to environmental cues such as diet, photoperiod, or temperature”. Thus, it is possible that the different compounds identified in the effluvia of sexually-mature *A*. *ludens* and *A*. *obliqua* calling males influenced by larval origin could have been a result of the chemical environment the larvae developed in (i.e., fruit pulp), triggering in the adult intrinsic and plastic metabolic routes that yielded the additional, many times novel compounds in the odor bouquets. Given that the differences in the scent bouquets of, for example, males originating from *C. pubescens* (five compounds) and *S. lycopersicum* cv. ‘Saladette’ (six compounds) compared to those of *C. × paradisi* cv. ‘Marsh’ (12 compounds) or *P. guajava* (12 compounds) were very large, and that the two Solanaceous hosts generated adults with the least number of compounds in the scent blends, it is possible that this unique chemical environment the larvae experienced caused the opposite effect, that is, it inhibited synthesis routes. Key to unraveling this in future studies will be the ancestral host *C*. *edulis*, from which adults originated that also produced odor bouquets with few (seven) compounds.

With regard to the antibiotics treatment, we discovered a consistent trend indicating that the cocktail of antibiotics fed to adults (streptomycin and rifampicin) impinged on the quantity of scent bouquet components produced, but not the type of compounds in the blend. With the exception of (*Z,Z*)-3,6-nonadien-1-ol in flies from peach and epianastrephin in flies from artificial diets, flies treated with the antibiotics produced significantly less scent bouquet than untreated ones (Figure 4). However, despite the lack of significance found for these compounds, the trend is consistent, indicating a reduction in the concentration of the compounds influenced by the antibiotic’s treatment. Possibly increasing sample size would lead to finding statistically significant results for these compounds in future studies. The strongest effect was observed in the case of laboratory-reared flies (since they produced higher amounts of the scent bouquet than wild flies), but the phenomenon was also recorded in flies originating from peaches (Figure 4). Our results related to the higher amount of scent bouquet in flies stemming from an artificial diet compared with flies originating from fruit are like the effect of domestication on the effluvia profiles of *Bactrocera tryoni* (Froggat) males [85]. The scent bouquet production of *A. ludens* and *A. obliqua* males was also higher in flies fed with diets containing sugar: yeast (3:1) compared to those fed on fruit or fruit juice or only sugar [41]. Tephritid flies are known to have a close ecological relationship with bacteria [86,87,88,89,90,91], and it is possible that the reduction in the concentration of the scent bouquet compounds observed in flies treated with antibiotics was the result of drastically reducing the bacteria in the gut of the adult flies treated with them. Liedo et al. [41] evaluated the effect of post-teneral diets on the mating performance and pheromone production of sterile flies, and suggested the influence of bacteria, nutraceutics, or semiochemicals. Based on literature reports on the role of bacteria in sex pheromone production in insects, it is likely that in *A. ludens* this is also the case. This could explain the patterns observed in Figure 4, where the scent bouquet composition in terms of the compounds that form the odor blend did not change between flies treated and not treated with antibiotics, but the amounts were drastically reduced. Alternatively, if bacteria play a role in other critical metabolic routes but not in the synthesis of the sex pheromone, a tradeoff could have taken place where critical aspects for fly survival were privileged over scent bouquet production, and this could explain the very low levels of scent bouquet in treated flies compared to the amounts released by sexually-mature calling males with intact gut microbiota.

The “core scent bouquet” was conserved independently of the host fruit in which larvae developed, even in flies stemming from an artificial diet. Thus, it is likely that the genes encoding for the synthesis of these components are part of the biochemical machinery of the flies. In the case of novel compounds, it is possible that they could be sequestered/metabolized from fruit or provided by bacteria related to the fruit. Alternatively, these novel compounds required the activation of additional genes encoding for the novel synthesis routes. This could be the prelude to a speciation process conferring competitive advantages to the populations harboring the new metabolic routes. Based on literature reports, we suggest possible synthesis routes of certain scent bouquet compounds and their possible relationship with bacteria as there is the possibility of a mixed strategy in which flies developed endogenous synthesis routes encoded by specific genes related to certain compounds over evolutionary time, and on the other hand, they could also rely on bacteria for the synthesis of the remaining compounds found in the effluvia (scent bouquet) of sexually-mature calling males (Supplementary Figure S1). This is a pertinent question as we found that flies stemming from a fruit never attacked in nature but that was infested under forced artificial conditions produced a compound (tentatively p-cymen-7-ol) never reported before in the known sex pheromone of *A. ludens* [51,52,53,54,55], and many others that were not found in what we here deemed the ancestral scent bouquet of *A. ludens*. When comparing the scent bouquet of flies stemming from guava with the one from flies stemming from White Sapote, there were 12 compounds in guava and seven in White Sapote (“core” scent bouquet). This is intriguing as the natural question is: how were these additional/novel compounds synthesized? Wybouw et al. [92] review the role horizontal gene transfer could have had on the evolution of arthropod herbivory including assimilation and detoxification of noxious metabolites in plants. Could it be that this functional diversification brought by the newly acquired genes encoding for traits facilitating the colonization of new hosts resulted in collateral benefits such as scent bouquet synthesis? Or is the synthesis of pheromonal components so costly that a multiway system evolved including the sequestration of precursors or complete pheromone components, synthesis *de novo* by the insects’ own metabolic routes and synthesis of additional components by bacteria? Based on literature, for example the volatile compounds α-farnesene, α-bergamotene, trans-sesquisabinene hydrate, and β-bisabolene could be synthesized in three stages by the Mevalonate pathway [10,93,94]: the first stage involves the construction of two compounds of five carbons (C5); the isopentyl diphosphate (IPP) formed from the pathway of the mevalonic acid used by plants and insects, and the dimethylalyl diphosphate (DMAPP) synthesized by the Methylerythritol phosphate/1-deoxylulose-5-phosphate (MEP/DOXP) pathway generally used by bacteria and also by plants [10,95,96,97,98,99]. The second stage involves the construction of geranyl diphosphate (initial compound for the biosynthesis of monoterpenes (C10)), based on the condensation of DMAPP by the enzyme geranyl synthase, and the third stage includes the formation of sesquiterpenes and is carried out by the farnesyl diphosphate synthase to produce farnesyl diphosphate (FPP) from isopentyl diphosphate and geranyl diphosphate [99,100,101,102]. Finally, the sesquiterpene synthase (TPS) can produce, catalyze, or transform the FPP into a wide variety of sesquiterpenes such as α-farnesene, α-bergamotene, trans-sesquisabinene hydrate, and β-bisabolene (Figure S1) [2,97,101,102].

In nature it is common for *A*. *ludens* to encounter two/three hosts with simultaneously ripening fruit, or with fruit ripening in close succession [47,103]. Therefore, it is likely that females will encounter calling males stemming from different hosts [47,48]. As documented here, the scent bouquet of flies stemming from ancestral, exotic, and conditional hosts is quite different and variable (Figure 1 and Figure 2), and this could lead to females queuing into males from a “novel host”. It could also happen that, due to extreme drought conditions, the ancestral and alternative hosts do not fruit or produce few fruit in a particular season, and as a result, a “novel” host is used by flies (e.g., Manzano pepper) [104]. The latter also has important implications for the sterile insect technique (SIT), as millions of sterile males are released in vast areas, encompassing various habitats. As documented here, the scent bouquet of males stemming from a laboratory colony reared on an artificial diet differs in both the number of compounds present and in the quantity of them, compared to the scent bouquet of males from natural hosts (e.g., *P. persica*). If sterile males are massively released in, for example, mango- or citrus- producing areas, it could happen that local females, adapted to the scent bouquet of local males originating from one or two hosts, will not be attracted to the odor blend of the mass-reared males. The new (“novel”) compounds we detected in the effluvia of sexually-mature calling males originating from, for example, guava or apples, could possibly enhance the attractiveness of the males releasing them. We will pursue these important questions in electroantennogram, wind tunnel, and field-cage behavioral studies.

## 5. Conclusions

Here we have documented a strong influence of the larval host of two polyphagous fruit flies (*A*. *ludens* and *A*. *obliqua*) on the scent bouquet of sexually-mature calling males in terms of the number of compounds that configure the odor blend and also in the concentration of certain blend components. That is, we confirmed the host-quality-effect hypothesis. When comparing what we defined as the “core” scent bouquet found in males from ancestral hosts with the scent bouquet of males from conditional hosts, the number of components in the odor bouquet almost doubled, and in some “novel” hosts, new, hitherto unreported compounds, appeared. It is likely that some odor blend precursors are sequestered by larvae from the fruit pulp and carried over to the adult passing through the pupal stage (metamorphosis) via fat cells, but this will require further examination. Alternatively, phenotypic plasticity may be acting. We surmise that if some or all the new odors play a role in mate attraction, this could increase male mating success and possibly drive speciation processes. From a practical perspective, this scenario could also influence the mating success of mass-reared, sterilized males released as part of pest management programs applying the sterile insect technique on an area-wide basis. With respect to the synthesis routes, it is possible that the adult flies resort to their endogenous machinery, that some compounds are synthesized by bacteria, or that both mechanisms are at play. This, plus the possibility that assortative mating could take place, requires additional research.

## Figures and Tables

**Table 1:** Description of treatments and origin of *Anastrepha ludens* and *A. obliqua* males used to collect the scent bouquets contained in the effluvia emanating from abdominal pouches and the proctiger of sexually-mature virgin calling males.

Fly Species	Plant Species	Plant Family	Type of Infestation
*A. ludens*	*Casimiroa edulis* ^1^	Rutaceae	Naturally infested
	*Citrus aurantium* ^2^	Rutaceae	Naturally infested
	*C. × paradisi* cv. ‘Marsh’ ^2^	Rutaceae	Naturally infested
	*Mangifera indica* cv. ‘Manila’ ^2,^^5^	Anacardiaceae	Laboratory forced infestation
	*Prunus persica* cv. ‘Criollo’ ^2^	Rosaseae	Forced field infestation
	*Pyrus communis* ^2^	Rosaceae	Naturally infested
	*Malus × domestica* cv. ‘Rayada’ ^2^	Rosaceae	Forced field infestation
	*Malus × domestica* cv. ‘Golden Delicious’ ^2^	Rosaceae	Forced field infestation
	*Punica granatum* ^2^	Lythraceae	Naturally infested
	*Capsicum pubescens* ^2^	Solanaceae	Forced field infestation
	*Solanum lycopersicum* cv. ‘Saladette’ ^3^	Solanaceae	Forced field infestation
	*Psidium guajava* cv. ‘Criolla’ ^3^	Myrtaceae	Forced field infestation
*A. obliqua*	*Spondias mombin* ^1^	Anacardiaceae	Naturally infested
	*M. indica* cv. ‘Manila’ ^2^	Anacardiaceae	Naturally infested
	*Spondias purpurea* ^4^	Anacardiaceae	Forced field infestation
	*S. lycopersicum* cv. ‘Saladette’ ^3^	Solanaceae	Forced field infestation
	*P. guajava* cv. ‘Criolla’ ^3^	Myrtaceae	Forced field infestation

^1^ Ancestral host, ^2^ exotic host, ^3^ non-natural host, ^4^ native host, ^5^ purchased in local supermarket.

## Supplementary Materials

The following are available online at https://www.mdpi.com/2075-4450/11/5/309/s1, Table S1. Details on the fruit collection sites and localities in Mexico where forced infestations were obtained; Table S2. Identification of volatile compounds in the effluvia (scent bouquet) released by sexually mature, calling *A. ludens* and *A. obliqua* males as a function of the host fruit in which the larvae developed. RT: Retention time in minutes (min); Table S3. Volatile compounds identified in the effluvia (scent bouquet) released by sexually mature, calling *Anastrepha ludens* adult males stemming from a laboratory colony and reared on an artificial diet or originating from *Prunus persica* fruit treated or not with antibiotics. Rt: Retention time in minutes (min); Table S4. Volatile compounds reported in the literature as being released by sexually mature, calling *Anastrepha ludens* and *A. obliqua* adult males; Figure S1. Potential biochemical routes involved in the synthesis of some scent bouquet components appearing in the effluvia of sexually mature, calling *Anastrepha ludens* and *A. obliqua* males originating from different host fruit; Supplementary File S1. This Supplementary File presents the multiple comparisons of means performed after ANOVA tests detected significant effects of the predictor variable (i.e., host fruit) in the response variables (i.e., concentrations of chemical compounds) (See the Materials and Methods and Results section of the main text for details). We present the output of the Tukey contrasts computed in the R software using the *glht* function of the package *multcomp* (Hothorn et al. 2008). This R (R Development Core Team 2017) output present the common names of host fruit as follows: *Citrus aurantium* = Bitter Orange; *C. × paradisi* cv. ‘Marsh’ = Grapefruit; *Casimiroa edulis* = White Sapote; *Mangifera indica* cv. ‘Manila’ = Mango; *Prunus persica* cv. ‘Criollo’ = Peach; *Solanum lycopersicum* cv. ‘Saladette’ = Tomato; *Capsicum pubescens* = Manzano Pepper; *Psidium guajava* cv. ‘Criolla’ = Guava; *Spondias mombin* = Jobo; *S. purpurea* = Tropical Plum.
