# Supplementary material for: Host Plant and Antibiotic Effects on Scent Bouquet Composition of Anastrepha ludens and Anastrepha obliqua Calling Males, Two Polyphagous Tephritid Pests
Source: Insects. 2020 May 14;11(5):309. doi: 10.3390/insects11050309 (PMC7290347; doi:10.3390/insects11050309)
Supplement: Supplementary file 1 [file insects-11-00309-s001.zip › Supplementary Figure.docx]

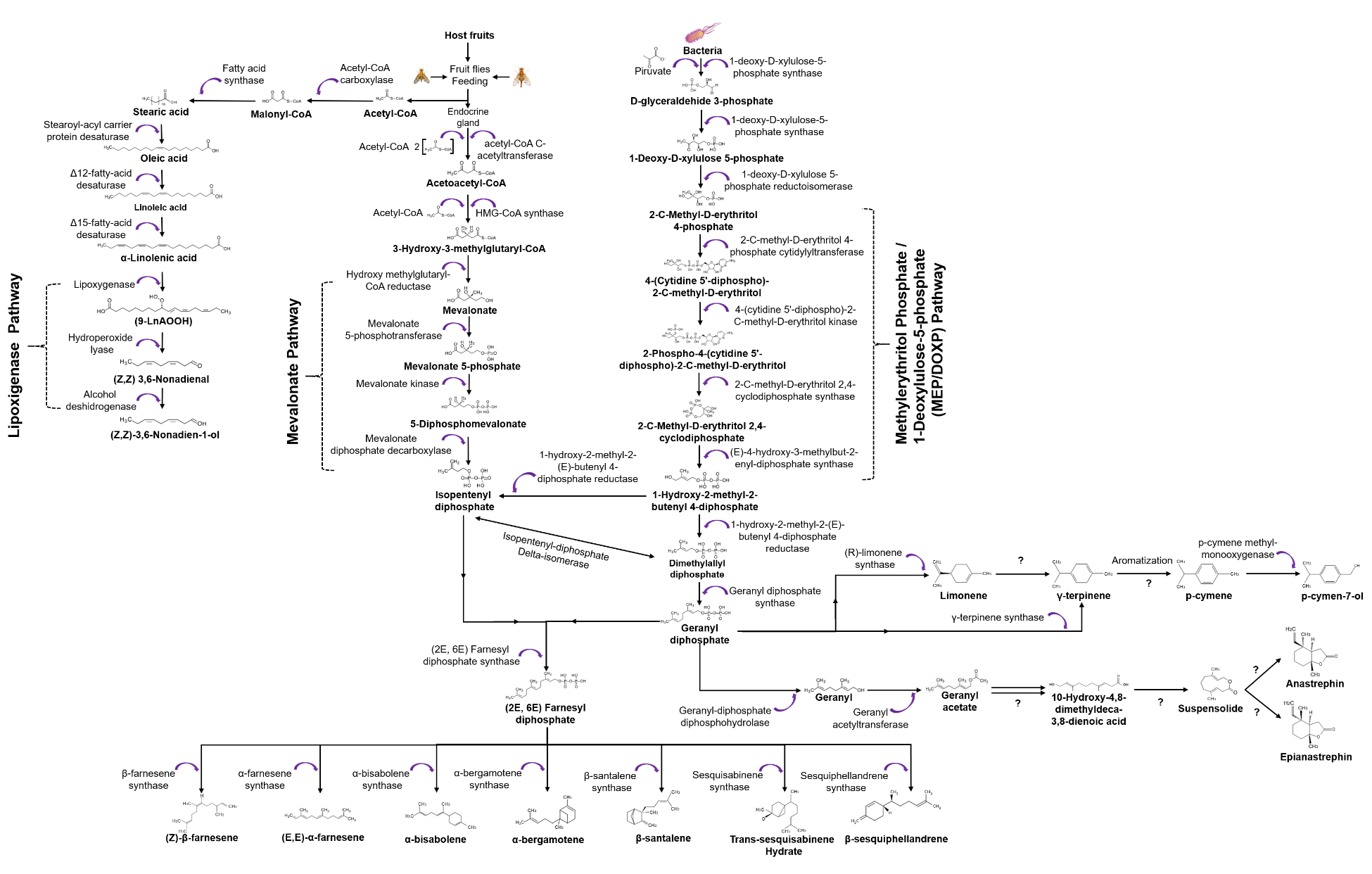


**Figure S1.** Potential biochemical routes involved in the synthesis of some scent bouquet components appearing in the effluvia of sexually mature, calling *Anastrepha ludens* and *A. obliqua* males originating from different host fruit.
