# Supplementary material for: Host Plant and Antibiotic Effects on Scent Bouquet Composition of Anastrepha ludens and Anastrepha obliqua Calling Males, Two Polyphagous Tephritid Pests
Source: Insects. 2020 May 14;11(5):309. doi: 10.3390/insects11050309 (PMC7290347; doi:10.3390/insects11050309)
Supplement: Supplementary file 1 [file insects-11-00309-s001.zip › Supplementary File.docx]

**Supplementary File S1**

**Host plant and antibiotic effects on scent bouquet composition of *Anastrepha ludens* and *Anastrepha obliqua* calling males, two polyphagous tephritid pests**

**Martín Aluja^1*^, Gabriela Cabagne^1^, Alma Altúzar-Molina^1^, Carlos Pascacio-Villafán^1^, Erick Enciso^1^, and Larissa Guillén^1^**

1. Instituto de Ecología, A.C. - INECOL, Red de Manejo Biorracional de Plagas y Vectores, Clúster Científico y Tecnológico BioMimic^®^, Carretera antigua a Coatepec 351, 91073 Xalapa, Veracruz, Mexico; gabriela.cabagne@inecol.mx; alma.altuzar@inecol.mx; carlos.pascacio@inecol.mx; erick.enciso@inecol.mx; [larissa.guillen@inecol.mx](mailto:larissa.guillen@inecol.mx)

***** Correspondence: martin.aluja@inecol.mx; Tel.: +52 2288421841

This Supplementary File presents the multiple comparisons of means performed after ANOVA tests detected significant effects of the predictor variable (i.e., host fruit) in the response variables (i.e., concentrations of chemical compounds) (See the Materials and Methods and Results section of the main text for details). We present the output of the Tukey contrasts computed in the R software using the *glht* function of the package *multcomp* (Hothorn et al. 2008). This R (R Development Core Team 2017) output present the common names of host fruit as follows: *Citrus* *aurantium* = Bitter Orange; *C. × paradisi* cv. ʹMarshʹ = Grapefruit; *Casimiroa* *edulis* = White Sapote; *Mangifera indica* cv. ʹManilaʹ = Mango; *Prunus* *persica* cv. ʹCriolloʹ = Peach; *Solanum* *lycopersicum* cv. ʹSaladetteʹ = Tomato; *Capsicum* *pubescens* = Manzano Pepper; *Psidium* *guajava* cv. ʹCriollaʹ = Guava; *Spondias mombin* = Jobo; *S. purpurea* = Tropical Plum.

**Fly species:** *Anastrepha ludens*

**Compound:** (Z,Z)-3,6-nonadien-1-ol

Simultaneous Tests for General Linear Hypotheses

Multiple Comparisons of Means: Tukey Contrasts

Fit: lm(formula = log(X.Z.Z..3.6.nonadien.1.ol) ~ Host)

Linear Hypotheses:

Estimate Std. Error t value Pr(>|t|)

Grapefruit - Bitter orange == 0 0.966577 0.445671 2.169 0.3912

Guava - Bitter orange == 0 0.352645 0.445671 0.791 0.9927

Mango - Bitter orange == 0 0.349739 0.445671 0.785 0.9930

Manzano pepper - Bitter orange == 0 -2.826363 0.445671 -6.342 <0.001 ***

Peach - Bitter orange == 0 0.517485 0.445671 1.161 0.9382

Tomato - Bitter orange == 0 -2.936600 0.445671 -6.589 <0.001 ***

White sapote - Bitter orange == 0 -0.648578 0.445671 -1.455 0.8256

Guava - Grapefruit == 0 -0.613933 0.445671 -1.378 0.8618

Mango - Grapefruit == 0 -0.616839 0.445671 -1.384 0.8590

Manzano pepper - Grapefruit == 0 -3.792940 0.445671 -8.511 <0.001 ***

Peach - Grapefruit == 0 -0.449092 0.445671 -1.008 0.9706

Tomato - Grapefruit == 0 -3.903177 0.445671 -8.758 <0.001 ***

White sapote - Grapefruit == 0 -1.615155 0.445671 -3.624 0.0167 *

Mango - Guava == 0 -0.002906 0.445671 -0.007 1.0000

Manzano pepper - Guava == 0 -3.179007 0.445671 -7.133 <0.001 ***

Peach - Guava == 0 0.164840 0.445671 0.370 0.9999

Tomato - Guava == 0 -3.289244 0.445671 -7.380 <0.001 ***

White sapote - Guava == 0 -1.001222 0.445671 -2.247 0.3473

Manzano pepper - Mango == 0 -3.176102 0.445671 -7.127 <0.001 ***

Peach - Mango == 0 0.167746 0.445671 0.376 0.9999

Tomato - Mango == 0 -3.286338 0.445671 -7.374 <0.001 ***

White sapote - Mango == 0 -0.998317 0.445671 -2.240 0.3509

Peach - Manzano pepper == 0 3.343848 0.445671 7.503 <0.001 ***

Tomato - Manzano pepper == 0 -0.110237 0.445671 -0.247 1.0000

White sapote - Manzano pepper == 0 2.177785 0.445671 4.887 <0.001 ***

Tomato - Peach == 0 -3.454084 0.445671 -7.750 <0.001 ***

White sapote - Peach == 0 -1.166063 0.445671 -2.616 0.1799

White sapote - Tomato == 0 2.288022 0.445671 5.134 <0.001 ***

---

Signif. codes: 0 ‘***’ 0.001 ‘**’ 0.01 ‘*’ 0.05 ‘.’ 0.1 ‘ ’ 1

(Adjusted p values reported -- single-step method)

**Fly species:** *Anastrepha ludens*

**Compound:** Epianastrephin

Simultaneous Tests for General Linear Hypotheses

Multiple Comparisons of Means: Tukey Contrasts

Fit: lm(formula = 1/sqrt(epianastrephin) ~ Host)

Linear Hypotheses:

Estimate Std. Error t value Pr(>|t|)

Grapefruit - Bitter orange == 0 -0.10429 0.04514 -2.310 0.313

Guava - Bitter orange == 0 -0.08469 0.04514 -1.876 0.575

Mango - Bitter orange == 0 -0.03522 0.04514 -0.780 0.993

Manzano pepper - Bitter orange == 0 0.42498 0.04514 9.415 <0.001 ***

Peach - Bitter orange == 0 -0.06512 0.04514 -1.443 0.832

Tomato - Bitter orange == 0 0.39262 0.04514 8.698 <0.001 ***

White sapote - Bitter orange == 0 0.03688 0.04514 0.817 0.991

Guava - Grapefruit == 0 0.01960 0.04514 0.434 1.000

Mango - Grapefruit == 0 0.06906 0.04514 1.530 0.787

Manzano pepper - Grapefruit == 0 0.52927 0.04514 11.726 <0.001 ***

Peach - Grapefruit == 0 0.03917 0.04514 0.868 0.987

Tomato - Grapefruit == 0 0.49690 0.04514 11.009 <0.001 ***

White sapote - Grapefruit == 0 0.14117 0.04514 3.128 0.059 .

Mango - Guava == 0 0.04946 0.04514 1.096 0.954

Manzano pepper - Guava == 0 0.50967 0.04514 11.292 <0.001 ***

Peach - Guava == 0 0.01957 0.04514 0.434 1.000

Tomato - Guava == 0 0.47730 0.04514 10.575 <0.001 ***

White sapote - Guava == 0 0.12157 0.04514 2.693 0.154

Manzano pepper - Mango == 0 0.46020 0.04514 10.196 <0.001 ***

Peach - Mango == 0 -0.02989 0.04514 -0.662 0.998

Tomato - Mango == 0 0.42784 0.04514 9.479 <0.001 ***

White sapote - Mango == 0 0.07211 0.04514 1.598 0.749

Peach - Manzano pepper == 0 -0.49009 0.04514 -10.858 <0.001 ***

Tomato - Manzano pepper == 0 -0.03236 0.04514 -0.717 0.996

White sapote - Manzano pepper == 0 -0.38810 0.04514 -8.598 <0.001 ***

Tomato - Peach == 0 0.45773 0.04514 10.141 <0.001 ***

White sapote - Peach == 0 0.10200 0.04514 2.260 0.340

White sapote - Tomato == 0 -0.35573 0.04514 -7.881 <0.001 ***

---

Signif. codes: 0 ‘***’ 0.001 ‘**’ 0.01 ‘*’ 0.05 ‘.’ 0.1 ‘ ’ 1

(Adjusted p values reported -- single-step method)

**Fly species:** *Anastrepha ludens*

**Compound:** Anastrephin

Simultaneous Tests for General Linear Hypotheses

Multiple Comparisons of Means: Tukey Contrasts

Fit: lm(formula = 1/sqrt(anastrephin) ~ Host)

Linear Hypotheses:

Estimate Std. Error t value Pr(>|t|)

Grapefruit - Bitter orange == 0 -0.196016 0.071575 -2.739 0.142

Guava - Bitter orange == 0 -0.146173 0.071575 -2.042 0.468

Mango - Bitter orange == 0 -0.098672 0.071575 -1.379 0.861

Manzano pepper - Bitter orange == 0 0.594804 0.071575 8.310 <0.001 ***

Peach - Bitter orange == 0 -0.103843 0.071575 -1.451 0.827

Tomato - Bitter orange == 0 0.457351 0.071575 6.390 <0.001 ***

White sapote - Bitter orange == 0 0.006621 0.080023 0.083 1.000

Guava - Grapefruit == 0 0.049843 0.071575 0.696 0.997

Mango - Grapefruit == 0 0.097344 0.071575 1.360 0.869

Manzano pepper - Grapefruit == 0 0.790820 0.071575 11.049 <0.001 ***

Peach - Grapefruit == 0 0.092173 0.071575 1.288 0.897

Tomato - Grapefruit == 0 0.653367 0.071575 9.128 <0.001 ***

White sapote - Grapefruit == 0 0.202637 0.080023 2.532 0.213

Mango - Guava == 0 0.047500 0.071575 0.664 0.997

Manzano pepper - Guava == 0 0.740977 0.071575 10.353 <0.001 ***

Peach - Guava == 0 0.042330 0.071575 0.591 0.999

Tomato - Guava == 0 0.603523 0.071575 8.432 <0.001 ***

White sapote - Guava == 0 0.152794 0.080023 1.909 0.552

Manzano pepper - Mango == 0 0.693477 0.071575 9.689 <0.001 ***

Peach - Mango == 0 -0.005170 0.071575 -0.072 1.000

Tomato - Mango == 0 0.556023 0.071575 7.768 <0.001 ***

White sapote - Mango == 0 0.105293 0.080023 1.316 0.887

Peach - Manzano pepper == 0 -0.698647 0.071575 -9.761 <0.001 ***

Tomato - Manzano pepper == 0 -0.137454 0.071575 -1.920 0.545

White sapote - Manzano pepper == 0 -0.588183 0.080023 -7.350 <0.001 ***

Tomato - Peach == 0 0.561193 0.071575 7.841 <0.001 ***

White sapote - Peach == 0 0.110464 0.080023 1.380 0.860

White sapote - Tomato == 0 -0.450730 0.080023 -5.633 <0.001 ***

---

Signif. codes: 0 ‘***’ 0.001 ‘**’ 0.01 ‘*’ 0.05 ‘.’ 0.1 ‘ ’ 1

(Adjusted p values reported -- single-step method)

**Fly species:** *Anastrepha ludens*

**Compound:** (E,E)-α-farnesene

Simultaneous Tests for General Linear Hypotheses

Multiple Comparisons of Means: Tukey Contrasts

Fit: lm(formula = log(X.E.E..alpha.Farnesene) ~ Host)

Linear Hypotheses:

Estimate Std. Error t value Pr(>|t|)

Grapefruit - Bitter orange == 0 0.57284 0.38629 1.483 0.75028

Guava - Bitter orange == 0 0.53062 0.38629 1.374 0.80950

Mango - Bitter orange == 0 -0.72939 0.38629 -1.888 0.50010

Peach - Bitter orange == 0 -0.80273 0.38629 -2.078 0.38736

Tomato - Bitter orange == 0 -2.53589 0.47310 -5.360 < 0.001 ***

White sapote - Bitter orange == 0 -1.68913 0.40514 -4.169 0.00370 **

Guava - Grapefruit == 0 -0.04223 0.38629 -0.109 1.00000

Mango - Grapefruit == 0 -1.30223 0.38629 -3.371 0.02907 *

Peach - Grapefruit == 0 -1.37558 0.38629 -3.561 0.01826 *

Tomato - Grapefruit == 0 -3.10873 0.47310 -6.571 < 0.001 ***

White sapote - Grapefruit == 0 -2.26197 0.40514 -5.583 < 0.001 ***

Mango - Guava == 0 -1.26001 0.38629 -3.262 0.03811 *

Peach - Guava == 0 -1.33335 0.38629 -3.452 0.02409 *

Tomato - Guava == 0 -3.06650 0.47310 -6.482 < 0.001 ***

White sapote - Guava == 0 -2.21974 0.40514 -5.479 < 0.001 ***

Peach - Mango == 0 -0.07334 0.38629 -0.190 1.00000

Tomato - Mango == 0 -1.80650 0.47310 -3.818 0.00965 **

White sapote - Mango == 0 -0.95973 0.40514 -2.369 0.24330

Tomato - Peach == 0 -1.73316 0.47310 -3.663 0.01420 *

White sapote - Peach == 0 -0.88639 0.40514 -2.188 0.32873

White sapote - Tomato == 0 0.84676 0.48862 1.733 0.59777

---

Signif. codes: 0 ‘***’ 0.001 ‘**’ 0.01 ‘*’ 0.05 ‘.’ 0.1 ‘ ’ 1

(Adjusted p values reported -- single-step method)

**Fly species:** *Anastrepha obliqua*

**Compound:** (Z,Z)-3,6-nonadien-1-ol

Simultaneous Tests for General Linear Hypotheses

Multiple Comparisons of Means: Tukey Contrasts

Fit: lm(formula = 1/sqrt(X.Z.Z..3.6.nonadien.1.ol) ~ Host)

Linear Hypotheses:

Estimate Std. Error t value Pr(>|t|)

jobo - Guava == 0 -0.03319 0.02117 -1.568 0.53100

Mango - Guava == 0 -0.08728 0.02117 -4.123 0.00318 **

Tomato - Guava == 0 -0.01237 0.02220 -0.557 0.97995

Tropical plum - Guava == 0 -0.06137 0.02117 -2.899 0.05525 .

Mango - jobo == 0 -0.05410 0.02117 -2.555 0.11137

Tomato - jobo == 0 0.02082 0.02220 0.938 0.87930

Tropical plum - jobo == 0 -0.02819 0.02117 -1.331 0.67487

Tomato - Mango == 0 0.07491 0.02220 3.374 0.01935 *

Tropical plum - Mango == 0 0.02591 0.02117 1.224 0.73774

Tropical plum - Tomato == 0 -0.04900 0.02220 -2.207 0.21093

---

Signif. codes: 0 ‘***’ 0.001 ‘**’ 0.01 ‘*’ 0.05 ‘.’ 0.1 ‘ ’ 1

(Adjusted p values reported -- single-step method)

**Fly species:** *Anastrepha obliqua*

**Compound:** (Z)-β-farnesene

Simultaneous Tests for General Linear Hypotheses

Multiple Comparisons of Means: Tukey Contrasts

Fit: lm(formula = ((X.Z..beta.farnesene)^-1.69) ~ Host)

Linear Hypotheses:

Estimate Std. Error t value Pr(>|t|)

jobo - Guava == 0 0.21241 0.09671 2.196 0.21495

Mango - Guava == 0 -0.14598 0.09671 -1.509 0.56642

Tomato - Guava == 0 0.09275 0.10143 0.914 0.88848

Tropical plum - Guava == 0 -0.11756 0.09671 -1.216 0.74249

Mango - jobo == 0 -0.35839 0.09671 -3.706 0.00882 **

Tomato - jobo == 0 -0.11966 0.10143 -1.180 0.76242

Tropical plum - jobo == 0 -0.32997 0.09671 -3.412 0.01764 *

Tomato - Mango == 0 0.23873 0.10143 2.354 0.16293

Tropical plum - Mango == 0 0.02842 0.09671 0.294 0.99825

Tropical plum - Tomato == 0 -0.21031 0.10143 -2.073 0.26359

---

Signif. codes: 0 ‘***’ 0.001 ‘**’ 0.01 ‘*’ 0.05 ‘.’ 0.1 ‘ ’ 1

(Adjusted p values reported -- single-step method)

**References**

Hothorn, T.; Bretz, F.; Westfall, P. Simultaneous inference in general parametric models. *Biom. J.* **2008**, *50*, 346–363.

R Development Core Team. *R: A Language and Environment for Statistical Computing*; R Foundation for Statistical Computing: Vienna, Austria, 2017.
