# Supplementary material for: Host Plant and Antibiotic Effects on Scent Bouquet Composition of Anastrepha ludens and Anastrepha obliqua Calling Males, Two Polyphagous Tephritid Pests
Source: Insects. 2020 May 14;11(5):309. doi: 10.3390/insects11050309 (PMC7290347; doi:10.3390/insects11050309)
Supplement: Supplementary file 1 [file insects-11-00309-s001.zip › Supplementary Tables.docx]

**Table S1.** Details on the fruit collection sites and localities in Mexico where forced infestations were obtained.

| **Fly species** | **Plant species** | **Type of infestation** | **Location** | **Municipality** | **State** | **Latitude** | **Longitude** | **m.a.s.l.** |
| --- | --- | --- | --- | --- | --- | --- | --- | --- |
| *A. ludens* | *C. edulis* | Naturally infested | Xalapa | Xalapa | Veracruz | 19°32'14.64"N | 96°54'4.95"W | 1374 |
| *A. ludens* | *C. aurantium* | Naturally infested | Tuzamapan | Coatepec | Veracruz | 19°24'10.25"N | 96°52'56.32"W | 921 |
| *A. ludens* | *C. × paradisi* cv. ‘Marsh’ | Naturally infested | Alborada | Coatepec | Veracruz | 19°26'28.14"N | 96°53'28.37"W | 1096 |
| *A. ludens* | *M. indica* cv. ‘Manila’ | Laboratory forced infestation | Semi-wild colony | - | - | - | - | - |
| *A. ludens* | *P. persica* cv. ‘Criollo’ | Forced field infestation | Xalapa | Xalapa | Veracruz | 19°33'45.29"N | 96°54'19.80"W | 1368 |
| *A. ludens* | *P. communis* | Naturally infested | Durango | Zimapán | Hidalgo | 20° 53'36.58"N | 99° 13'54.47"W | 2115 |
| *A. ludens* | *M. × domestica* cv. ‘Golden Delicious’ | Forced field infestation | Durango | Zimapán | Hidalgo | 20° 53'36.58"N | 99° 13'54.47"W | 2115 |
| *A. ludens* | *M. × domestica* cv. ‘Rayada’ | Forced field infestation | Durango | Zimapán | Hidalgo | 20° 53'36.58"N | 99° 13'54.47"W | 2115 |
| *A. ludens* | *P. granatum* | Naturally infested | Tasquillo | Tasquillo | Hidalgo | 20° 32'41.52"N | 99° 21'16.85"W | 1659 |
| *A. ludens* | *C. pubescens* | Forced field infestation | Cuauhtemecatla | Xico | Veracruz | 19°27'21.09"N | 97° 5'3.94"W | 2267 |
| *A. ludens* | *S. lycopersicum* cv. ‘Saladette’ | Forced field infestation | El Ojite | Actopan | Veracruz | 19°25'9.65"N | 96°30'54.98"W | 90 |
| *A. ludens* | *P. guajava* | Forced field infestation | Jilotepec | Jilotepec | Veracruz | 19°36'13.79"N | 96°57'29.25"W | 1449 |
| *A. obliqua* | *S. mombin* | Naturally infested | Alborada | Coatepec | Veracruz | 19°25'7.57"N | 96°52'19.45"W | 956 |
| *A. obliqua* | *M. indica* cv. ‘Manila’ | Naturally infested | Idolos | Actopan | Veracruz | 19°26'20.31"N | 96°30'52.70"W | 105 |
| *A. obliqua* | *S. purpurea* | Forced field infestation | El Palmar | Puente Nacional | Veracruz | 19°22'43.06"N | 96°25'15.33""W | 32 |
| *A. obliqua* | *S. lycopersicum* cv. ‘Saladette’ | Forced field infestation | El Ojite | Actopan | Veracruz | 19°25'9.65"N | 96°30'54.98"W | 90 |
| *A. obliqua* | *P. guajava* | Forced field infestation | Jilotepec | Jilotepec | Veracruz | 19°36'13.79"N | 96°57'29.25"W | 1449 |

m.a.s.l.: meters above sea level

**Table S2.** Identification of volatile compounds in the effluvia (scent bouquet) released by sexually mature, calling *A. ludens* and *A. obliqua* males as a function of the host fruit in which the larvae developed. RT: Retention time in minutes (min).

| Fly species | Fruit specie | Volatile compound | Rt (min) | Reproducibility | Abundance (peak area/1 million) |
| --- | --- | --- | --- | --- | --- |
| *A. ludens* | *C. edulis* | (*Z,Z*)-3,6-nonadien-1-ol | 11.80 | 6/6 | 0.9464 |
|  |  | α-bergamotene | 14.70 | 6/6 | 0.2064 |
|  |  | 1-cyclopentanecarboxylic acid, 4-isopropylidene-2-vinyl-, methyl ester, cis | 15.07 | 4/6 | 0.2009 |
|  |  | (*E,E*)-α-farnesene | 15.23 | 6/6 | 0.1169 |
|  |  | suspensolide | 15.27 | 6/6 | 0.7385 |
|  |  | anastrephin | 16.09 | 6/6 | 0.2691 |
|  |  | epianastrephin | 16.19 | 6/6 | 0.3608 |
| *A. ludens* | *C. pubescens* | (*Z,Z*)-3,6-nonadien-1-ol | 11.80 | 5/6 | 0.1434 |
|  |  | α-bergamotene | 14.70 | 4/6 | 0.1583 |
|  |  | suspensolide | 15.27 | 6/6 | 1.0852 |
|  |  | anastrephin | 16.09 | 5/6 | 0.1684 |
|  |  | epianastrephin | 16.19 | 5/6 | 0.2222 |
| *A. ludens* | *S. lycopersicum* | (*Z,Z*)-3,6-nonadien-1-ol | 11.80 | 4/6 | 0.0802 |
|  |  | α-bergamotene | 14.70 | 6/6 | 0.1462 |
|  |  | (*E,E*)-α-farnesene | 15.23 | 3/6 | 0.0422 |
|  |  | suspensolide | 15.27 | 6/6 | 1.0178 |
|  |  | anastrephin | 16.09 | 5/6 | 0.1507 |
|  |  | epianastrephin | 16.19 | 5/6 | 0.1656 |
| *A. ludens* | *M. indica* | (*Z,Z*)-3,6-nonadien-1-ol | 11.80 | 6/6 | 2.2553 |
|  |  | α-bergamotene | 14.70 | 6/6 | 0.2443 |
|  |  | 1-cyclopentanecarboxylic acid, 4-isopropylidene-2-vinyl-, methyl ester, cis | 15.07 | 4/6 | 0.2264 |
|  |  | (*E,E*)-α-farnesene | 15.23 | 6/6 | 0.6851 |
|  |  | suspensolide | 15.27 | 6/6 | 1.8863 |
|  |  | β-bisabolene | 15.30 | 3/6 | 0.0688 |
|  |  | anastrephin | 16.09 | 6/6 | 0.5006 |
|  |  | epianastrephin | 16.19 | 6/6 | 1.7471 |
| *A. ludens* | *P. persica* | (*Z,Z*)-3,6-nonadien-1-ol | 11.80 | 6/6 | 2.397 |
|  |  | α-bergamotene | 14.70 | 6/6 | 0.1636 |
|  |  | 1-cyclopentanecarboxylic acid, 4-isopropylidene-2-vinyl-, methyl ester, cis | 15.07 | 6/6 | 0.2658 |
|  |  | (*E,E*)-α-farnesene | 15.23 | 6/6 | 0.4767 |
|  |  | suspensolide | 15.27 | 6/6 | 0.8302 |
|  |  | bicyclo[5.2.0]nonane, 4-methylene-2,8,8-trimethyl-2-vinyl- | 15.52 | 4/6 | 0.0834 |
|  |  | anastrephin | 16.09 | 6/6 | 0.6395 |
|  |  | epianastrephin | 16.19 | 6/6 | 1.7828 |
| *A. ludens* | *M. domestica* cv. 'Golden Delicious*'* | (*Z*)-3-nonen-1-ol | 11.17 | 6/6 | 2.2706 |
|  |  | (*Z,Z*)-3,6-nonadien-1-ol | 11.20 | 6/6 | 1.1739 |
|  |  | α-bergamotene | 14.04 | 6/6 | 0.7603 |
|  |  | β-sesquiphellandrene | 14.10 | 4/6 | 0.1064 |
|  |  | β-santalene | 14.29 | 6/6 | 0.1006 |
|  |  | (*E,E*)-α-farnesene | 14.59 | 6/6 | 3.7302 |
|  |  | suspensolide | 14.63 | 6/6 | 7.1347 |
|  |  | β-bisabolene | 14.67 | 6/6 | 0.2743 |
|  |  | anastrephin | 15.40 | 6/6 | 1.8815 |
|  |  | epianastrephin | 15.51 | 6/6 | 6.3802 |
| *A. ludens* | *M. domestica* cv.ʹRayadaʹ | (*Z*)-3-nonen-1-ol | 11.17 | 6/6 | 1.9047 |
|  |  | (*Z,Z*)-3,6-nonadien-1-ol | 11.20 | 6/6 | 0.7332 |
|  |  | α-bergamotene | 14.04 | 6/6 | 0.4667 |
|  |  | β-sesquiphellandrene | 14.10 | 3/6 | 0.1256 |
|  |  | β-santalene | 14.29 | 3/6 | 0.1223 |
|  |  | (*E,E*)-α-farnesene | 14.59 | 6/6 | 2.0711 |
|  |  | suspensolide | 14.63 | 6/6 | 4.1097 |
|  |  | β-bisabolene | 14.67 | 4/6 | 0.181 |
|  |  | anastrephin | 15.40 | 6/6 | 0.9646 |
|  |  | epianastrephin | 15.51 | 6/6 | 3.3409 |
| *A. ludens* | *C. × aurantium* | (*Z*)-3-nonen-1-ol | 11.78 | 3/6 | 0.0948 |
|  |  | (*Z,Z*)-3-6-nonadienol | 11.80 | 5/6 | 2.5638 |
|  |  | α-bergamotene | 14.70 | 6/6 | 0.4434 |
|  |  | β-santalene | 14.94 | 3/6 | 0.0549 |
|  |  | (*E,E*)-α-farnesene | 15.23 | 6/6 | 1.421 |
|  |  | β-bisabolene | 15.30 | 2/6 | 0.2283 |
|  |  | suspensolide | 15.27 | 5/6 | 4.035 |
|  |  | bicyclo[5.2.0]nonane, 4-methylene-2,8,8-trimethyl-2-vinyl- | 15.52 | 4/6 | 0.1627 |
|  |  | anastrephin | 16.09 | 6/6 | 0.8467 |
|  |  | epianastrephin | 16.19 | 6/6 | 2.7454 |
| *A. ludens* | *P. communis* | (*Z*)-3-nonen-1-ol | 11.17 | 3/6 | 0.669 |
|  |  | (*Z,Z*)-3,6-nonadien-1-ol | 11.20 | 6/6 | 1.7312 |
|  |  | α-bergamotene | 14.04 | 6/6 | 0.6661 |
|  |  | β-sesquiphellandrene | 14.10 | 6/6 | 0.1015 |
|  |  | β-santalene | 14.29 | 6/6 | 0.0828 |
|  |  | 1-cyclopentanecarboxylic acid, 4-isopropylidene-2-vinyl-, methyl ester, cis | 14.38 | 4/6 | 0.0533 |
|  |  | (*E,E*)-α-farnesene | 14.59 | 6/6 | 1.8411 |
|  |  | suspensolide | 14.63 | 6/6 | 5.5944 |
|  |  | β-bisabolene | 14.67 | 6/6 | 0.2702 |
|  |  | anastrephin | 15.40 | 6/6 | 0.8787 |
|  |  | epianastrephin | 15.51 | 6/6 | 3.1866 |
| *A. ludens* | *P. granatum* | (*Z*)-3-nonen-1-ol | 11.17 | 5/6 | 1.1772 |
|  |  | (*Z,Z*)-3,6-nonadien-1-ol | 11.20 | 6/6 | 1.844 |
|  |  | α-bergamotene | 14.04 | 6/6 | 0.6886 |
|  |  | β-sesquiphellandrene | 14.10 | 4/6 | 0.0992 |
|  |  | β-santalene | 14.29 | 6/6 | 0.1282 |
|  |  | (*E,E*)-α-farnesene | 14.59 | 6/6 | 3.0956 |
|  |  | suspensolide | 14.63 | 6/6 | 6.0271 |
|  |  | β -bisabolene | 14.67 | 6/6 | 0.1825 |
|  |  | trans-sesquisabinene hydrate | 14.84 | 4/6 | 0.1174 |
|  |  | anastrephin | 15.40 | 6/6 | 1.1963 |
|  |  | epianastrephin | 15.51 | 6/6 | 4.2048 |
| *A. ludens* | *P. guajava* | (*Z*)-3-nonen-1-ol | 11.78 | 6/6 | 0.1316 |
|  |  | (*Z,Z*)-3,6-nonadien-1-ol | 11.80 | 6/6 | 1.8484 |
|  |  | p-cymen-7-ol | 12.84 | 4/6 | 0.2882 |
|  |  | α-bergamotene | 14.70 | 6/6 | 0.3473 |
|  |  | 1-cyclopentanecarboxylic acid, 4-isopropylidene-2-vinyl-, methyl ester, cis | 15.07 | 4/6 | 0.4577 |
|  |  | trans-sesquisabinene hydrate | 15.11 | 4/6 | 0.1153 |
|  |  | (*E,E*)-α-farnesene | 15.23 | 6/6 | 1.4174 |
|  |  | suspensolide | 15.27 | 6/6 | 1.3618 |
|  |  | β-bisabolene | 15.30 | 5/6 | 0.0793 |
|  |  | bicyclo[5.2.0]nonane, 4-methylene-2,8,8-trimethyl-2-vinyl- | 15.52 | 5/6 | 0.1487 |
|  |  | anastrephin | 16.09 | 6/6 | 0.8461 |
|  |  | epianastrephin | 16.19 | 6/6 | 1.9233 |
| *A. ludens* | *C. × paradisi* | (*Z*)-3-nonen-1-ol | 11.78 | 5/6 | 0.3197 |
|  |  | (*Z,Z*)-3,6-nonadien-1-ol | 11.80 | 6/6 | 5.2337 |
|  |  | α-bergamotene | 14.70 | 6/6 | 0.8234 |
|  |  | β-sesquiphellandrene | 14.75 | 3/6 | 0.1254 |
|  |  | β-santalene | 14.94 | 4/6 | 0.1175 |
|  |  | cyclopentanecarboxylic acid, 4-isopropylidene-2-vinyl-, methyl ester, cis | 15.07 | 6/6 | 1.0155 |
|  |  | (*E,E*)-α-farnesene | 15.23 | 6/6 | 2.8851 |
|  |  | suspensolide | 15.27 | 6/6 | 6.8787 |
|  |  | β-bisabolene | 15.30 | 4/6 | 0.1615 |
|  |  | bicyclo[5.2.0]nonane, 4-methylene-2,8,8-trimethyl-2-vinyl- | 15.52 | 6/6 | 0.4716 |
|  |  | anastrephin | 16.09 | 6/6 | 1.5628 |
|  |  | epianastrephin | 16.19 | 6/6 | 5.0779 |
| *A. obliqua* | *S. mombin* | (*Z,Z*)-3,6-nonadien-1-ol | 11.80 | 6/6 | 3.5167 |
|  |  | α-bergamotene | 15.11 | 6/6 | 8.9395 |
|  |  | (*E,E*)-α-farnesene | 15.23 | 6/6 | 4.0837 |
|  |  | tricyclo[3.1.0.0(2,4)]hexane, 3,6-diethyl-3,6-dimethyl-, trans- | 15.96 | 6/6 | 3.7601 |
| *A. obliqua* | *S. purpurea* | (*Z,Z*)-3,6-nnonadien-1-ol | 11.8 | 6/6 | 5.0319 |
|  |  | (*Z*) β-farnesene | 14.79 | 6/6 | 0.4366 |
|  |  | α-bergamotene | 15.11 | 6/6 | 20.2476 |
|  |  | (*E,E*)-α-farnesene | 15.23 | 6/6 | 5.9802 |
|  |  | β-bisabolene | 15.31 | 5/6 | 0.1871 |
|  |  | tricyclo[3.1.0.0(2,4)]hexane, 3,6-diethyl-3,6-dimethyl-, trans- | 15.96 | 5/6 | 8.8128 |
| *A. obliqua* | *S. lycopersicum* | (*Z,Z*)-3,6-nonadien-1-ol | 11.80 | 6/6 | 2.511 |
|  |  | (*Z*) β-farnesene | 14.79 | 6/6 | 0.2246 |
|  |  | α-bergamotene | 15.11 | 6/6 | 11.0635 |
|  |  | (*E,E*)-α-farnesene | 15.23 | 6/6 | 3.1317 |
|  |  | β -bisabolene | 15.31 | 3/6 | 0.1688 |
|  |  | tricyclo[3.1.0.0(2,4)]hexane, 3,6-diethyl-3,6-dimethyl-, trans | 15.96 | 6/6 | 3.7139 |
| *A. obliqua* | *M. indica* | (*Z,Z*)-3,6-nonadien-1-ol | 11.80 | 6/6 | 8.1258 |
|  |  | (*Z*) β-farnesene | 14.79 | 6/6 | 0.6198 |
|  |  | α-bergamotene | 15.11 | 6/6 | 24.5804 |
|  |  | (*E,E*)-α-farnesene | 15.23 | 6/6 | 7.8457 |
|  |  | β -bisabolene | 15.31 | 5/6 | 0.2555 |
|  |  | tricyclo[3.1.0.0(2,4)]hexane, 3,6-diethyl-3,6-dimethyl-, trans | 15.96 | 6/6 | 10.3849 |
| *A. obliqua* | *P. guajava* | (*Z*)-3-nonen-1-ol | 11.78 | 5/6 | 0.149 |
|  |  | (*Z,Z*)-3,6-nonadien-1-ol | 11.80 | 6/6 | 1.1753 |
|  |  | p-cymen-7-ol | 12.84 | 5/6 | 0.1302 |
|  |  | (*Z*) β-farnesene | 14.79 | 6/6 | 0.1006 |
|  |  | α-bergamotene | 15.11 | 6/6 | 8.5557 |
|  |  | (*E,E*)-α-farnesene | 15.23 | 6/6 | 2.5568 |
|  |  | β-bisabolene | 15.31 | 2/6 | 0.0863 |
|  |  | tricyclo[3.1.0.0(2,4)]hexane, 3,6-diethyl-3,6-dimethyl-, trans | 15.96 | 6/6 | 3.0407 |

**Table S3.** Volatile compounds identified in the effluvia (scent bouquet) released by sexually mature, calling *Anastrepha ludens* adult males stemming from a from laboratory colony reared on an artificial diet or originating from *Prunus persica* fruit treated or not with antibiotics*.* Rt: Retention time in minutes (min).

| **Treatment** | **Volatile compound** | **Rt (min)** | **Reproducibility** | **Abundance (peak area/ 1 million)** |
| --- | --- | --- | --- | --- |
| Laboratory colony flies | | | | |
| Without antibiotics | (Z)-3-nonen-1-ol | 11.78 | 6/6 | 2.953 |
|  | (Z,Z)-3,6-nonadien-1-ol | 11.80 | 6/6 | 4.4568 |
|  | α-bergamotene | 14.70 | 6/6 | 1.5586 |
|  | β-santalene | 14.94 | 6/6 | 0.1611 |
|  | 1-cyclopentanecarboxylic acid, 4-isopropylidene-2-vinyl-, methyl ester, cis | 15.07 | 6/6 | 1.7999 |
|  | bicyclo[5.2.0]nonane, 4-methylene-2,8,8-trimethyl-2-vinyl- | 15.11 | 6/6 | 0.5118 |
|  | (E,E)-α-farnesene | 15.23 | 6/6 | 13.0492 |
|  | suspensolide | 15.27 | 6/6 | 2.4962 |
|  | β-bisabolene | 15.30 | 6/6 | 0.2314 |
|  | tricyclo[3.1.0.0(2,4)]hexane,3,6-diethyl-3,6-dimethyl-, trans- | 15.96 | 6/6 | 0.2107 |
|  | anastrephin | 16.09 | 6/6 | 2.5926 |
|  | epianastrephin | 16.19 | 6/6 | 6.2655 |
| With antibiotics | (Z)-3-nonen-1-ol | 11.78 | 6/6 | 1.2059 |
|  | (Z, Z)-3,6-nonadien-1-ol | 11.80 | 6/6 | 2.6178 |
|  | α-bergamotene | 14.70 | 6/6 | 0.6512 |
|  | β-santalene | 14.90 | 4/6 | 0.0687 |
|  | 1-cyclopentanecarboxylic acid, 4-isopropylidene-2-vinyl-, methyl ester, cis | 15.07 | 6/6 | 0.6137 |
|  | bicyclo[5.2.0]nonane, 4-methylene-2,8,8-trimethyl-2-vinyl- | 15.16 | 6/6 | 0.1884 |
|  | (E,E)-α-farnesene | 15.23 | 6/6 | 5.0746 |
|  | suspensolide | 15.27 | 6/6 | 1.5865 |
|  | β-bisabolene | 15.30 | 2/6 | 0.0865 |
|  | tricyclo [3.1.0.0(2,4)] hexane, 3,6-diethyl-3,6-dimethyl-, trans | 15.96 | 5/6 | 0.0797 |
|  | anastrephin | 16.09 | 6/6 | 1.2525 |
|  | epianastrephin | 16.19 | 6/6 | 2.9962 |
| Flies from *P. persica* | | | | |
| Without antibiotics | (Z, Z)-3,6-nonadien-1-ol | 11.80 | 6/6 | 1.9872 |
|  | α -bergamotene | 14.70 | 6/6 | 0.1935 |
|  | 1-cyclopentanecarboxylic acid, 4-isopropylidene-2-vinyl-, methyl ester, cis | 15.07 | 6/6 | 0.2483 |
|  | (E,E)-α -farnesene | 15.23 | 6/6 | 0.4515 |
|  | suspensolide | 15.27 | 6/6 | 1.4844 |
|  | anastrephin | 16.09 | 6/6 | 0.5011 |
|  | epianastrephin | 16.19 | 6/6 | 1.4751 |
| With antibiotics | (Z, Z)-3,6-nonadien-1-ol | 11.80 | 6/6 | 1.1165 |
|  | α -bergamotene | 14.70 | 6/6 | 0.1918 |
|  | 1-cyclopentanecarboxylic acid, 4-isopropylidene-2-vinyl-, methyl ester, cis | 15.07 | 5/6 | 0.1564 |
|  | (E,E)-α -farnesene | 15.23 | 6/6 | 0.1853 |
|  | suspensolide | 15.27 | 6/6 | 1.3494 |
|  | anastrephin | 16.09 | 6/6 | 0.1942 |
|  | epianastrephin | 16.19 | 6/6 | 0.5842 |

**Table S4.** Volatile compounds reported in the literature as being released by sexually mature, calling *Anastrepha ludens* and *A. obliqua* adult males.

| **Compound** | **Host** | **Analysis technique** | **Reference** |
| --- | --- | --- | --- |
| *A. ludens* | | | |
| (*Z*)-3-nonenol | unknown | Cromatography on silver nitrate-impregnated silicic acid | Battiste, 1983 |
| (*Z,Z*)-3,6-nonadienol |  |  |  |
| anastrephin |  |  |  |
| epianastrephin |  |  |  |
| (*Z*)-3-nonenol | unknown | Liquid and gas chromatography | Stokes et al., 1983 |
| (*Z,Z*)-3,6-nonadienol |  |  |  |
| anastrephin |  |  |  |
| epianastrephin |  |  |  |
| (*Z*)-3-nonenol | laboratory | Tenax / Gas chromatography | Robacker and Hart, 1985; Robacker, 1988 |
| (*Z,Z*)-3,6-nonadienol |  |  |  |
| S,S-epianastrephin |  |  |  |
| (*Z*)-3-nonenol | unknown | Porapak Q adsortion/GC-MS analysis | Rocca et al. 1992 |
| (*Z,Z*)-3,6-nonadienol |  |  |  |
| anastrephin |  |  |  |
| epianastrephin |  |  |  |
| suspensolide |  |  |  |
| (*E,E*)-α-farnesene |  |  |  |
| β-bisabolene |  |  |  |
| α-trans-bergamotene |  |  |  |
| limonene |  |  |  |
| suspensolide | *Citrus aurantium* | Air‐entrainment technique/GC-MS | Liedo et al., 2013 |
| epianastrephin |  |  |  |
| anastrephin |  |  |  |
| (*E,E*)-α-farnesene |  |  |  |
| (*Z*)-3-nonenol | *C. edulis* and artificial diet | SPME/GC-MS | Quintero-Fong et al., 2016 |
| (*Z,Z*)-3,6-nonadienol |  |  |  |
| α-trans-bergamotene |  |  |  |
| (*E,Z*)- α-farnesene |  |  |  |
| (*E,E*)- α-farnesene |  |  |  |
| (*E,E*)-suspensolide |  |  |  |
| anastrephin |  |  |  |
| epianastrephin |  |  |  |
| (*Z,Z*)-3,6-nonadienol | *Citrus aurantium* and laboratory | SPME/GC-MS | Bosa et al., 2016 |
| (*Z*)-3-nonenol |  |  |  |
| (*E,E*)-suspensolide |  |  |  |
| epianastrephin |  |  |  |
| anastrephin |  |  |  |
| α-santalol |  |  |  |
| eudesm-7(11)-en-4-ol |  |  |  |
| (*E,E*)- α-farnesene |  |  |  |
| (*E,Z*)- α-farnesene |  |  |  |
| α-trans-bergamotene |  |  |  |
| ***A. obliqua*** | | | |
| (*E,E*)-α-farnesene | Unknown | Gas chromatography-mass spectrometry (GC/MS) | Ibañez-Lopez and Cruz-López, 2001 |
| (*Z,E*)- α-farnesene |  |  |  |
| (*Z*)-3-nonen-1-ol |  |  |  |
| (*E,E*)-α-farnesene | *Spondias mombin* | Gas chromatography-mass spectrometry (GC/MS) | López Guillén et al., 2008 |
| (*Z,E*)- α-farnesene |  |  |  |
| (*Z*)-3-nonen-1-ol |  |  |  |
| (*Z*)-3-nonenol | Unknown | Gas chromatography-mass spectrometry (GC/MS) | López-Guillén et al., 2011 |
| (*E,E*)-α-farnesene |  |  |  |
| (*Z,E*)-α-farnesene |  |  |  |
| nonadienol |  |  |  |
| β-farnesene |  |  |  |
| 3-hexanone | *Mangifera indica* (cv. Rosa) | Gas chromatography-mass spectrometry (GC/MS) | Goncalves et al., 2013 |
| 2-heptanone |  |  |  |
| 2,5-dimethylpyrazine |  |  |  |
| 2,6-dimethylpyrazyne |  |  |  |
| 2-methyl-4-heptanone |  |  |  |
| 4-methyl-3-heptanol |  |  |  |
| 1-heptanol |  |  |  |
| 3-octanone |  |  |  |
| 2-octanone |  |  |  |
| ethyl hexanoate |  |  |  |
| decane |  |  |  |
| methyl heptanoate |  |  |  |
| 2-ethylhexan-1-ol |  |  |  |
| limonene |  |  |  |
| (*Z*)-β-ocimene |  |  |  |
| 1-H-indene |  |  |  |
| 1-octanol |  |  |  |
| 3-ethyl-2,5-dimethylpyrazyne |  |  |  |
| linalool |  |  |  |
| ethyl heptanoate |  |  |  |
| methyl octanoate |  |  |  |
| (*E,Z*)-3,6-nonadien-1-ol |  |  |  |
| 1-nonanol |  |  |  |
| 1-phenylpropanone |  |  |  |
| 3-decanone |  |  |  |
| 2-decanone |  |  |  |
| ethyl octanoate |  |  |  |
| decanal |  |  |  |
| δ-elemene |  |  |  |
| α-copaene |  |  |  |
| geranyl acetate |  |  |  |
| β-elemene |  |  |  |
| (*E*)-β-caryophyllene |  |  |  |
| (*E*)-α-bergamotene |  |  |  |
| α-humulene |  |  |  |
| (*E,E*)-α-farnesene |  |  |  |
| (*Z,E*)-α-farnesene | *Spondias purpurea* | Gas chromatography-mass spectrometry (GC/MS) | Meza-Hernandez et al., 2002 |
| (*E,E*)-α-farnesene |  |  |  |
| (*Z*)-3-nonanol |  |  |  |
| (*Z*)-3-nonenol | *Spondias mombin* | Gas chromatography-mass spectrometry (GC/MS) | Muñoz Barrios et al., 2016 |
| (*Z,Z*)-3,6-nonadienol |  |  |  |
| (*Z,E*)-a-farnesene |  |  |  |
| (*E,E*)-a-farnesene |  |  |  |
| farnesene isomer |  |  |  |

**References**

Bosa, C.F.; Cruz‐López, L.; Zepeda‐Cisneros, C.S.; Valle‐Mora, J.; Guillén‐Navarro, K.; Liedo, P. Sexual behavior and male volatile compounds in wild and mass‐reared strains of the Mexican fruit fly *Anastrepha ludens* (Diptera: Tephritidae) held under different colony management regimes. *Insect Sci.* **2016**, *23,* 105-116.

Battiste, M.A.; Strekowski, L.; Vanderbilt, D.P.; Visnick, M.; King, R.W.; Nation, J.L. Anastrephin and epianastrephin, novel lactone components isolated from the sex pheromone blend of male Caribbean and Mexican fruit flies. *Tetrahedron Lett.* **1983**, *24*, 2611-2614.

Gonçalves, G.B.; Silva, C.E.; De Lima Mendonça, A.; Vaníčková, L.; Tomčala, A.; Nascimento, R.R.D. Pheromone communication in *Anastrepha obliqua* (Diptera: Tephritidae): A comparison of the volatiles and salivary gland extracts of two wild populations. *Fla. Entomol.* **2013**, *96*, 1365-1374.

Ibañez-López, A.; Cruz-López, L. Glándulas salivales de *Anastrepha obliqua* (Macquart) (Diptera: Tephritidae): análisis químico y morfológico, y actividad biológica de los componentes volátiles. *Folia Entomol. Mex.* **2001**, *40*, 221–231.

Liedo, P.; Orozco, D.; Cruz-López, L.; Quintero, J.L.; Becerra-Pérez, C.; Del Refugio-Hernández, M.; Oropeza, A.; Toledo, J. Effect of post-teneral diets on the performance of sterile *Anastrepha ludens* and *Anastrepha obliqua* fruit flies. *J. Appl. Entomol.* **2013,** *137*, 49-60.

López-Guillén, G.; Cruz-López, L.; Malo, E.A.; González-Hernández, H.; Llanderal-Cázares, C.; López-Collado, J.; Toledo, J.; Rojas, J.C. Factors influencing the release of volatiles in *Anastrepha obliqua* males (Diptera: Tephritidae). *Environ. Entomol.* **2008**, *37*, 876-882.

López-Guillén, G.; López, L.C.; Malo, E.A.; Rojas, J.C. Olfactory responses of *Anastrepha obliqua* (Diptera: Tephritidae) to volatiles emitted by calling males. *Fla. Entomol.* **2011**, *94*, 874-881.

Meza-Hernández, J.S.; Hernández, E.; Salvador-Figueroa, M.; Cruz-López, L. Sexual compatibility, mating performance and sex pheromone release of mass-reared and wild *Anastrepha obliqua* (Diptera: Tephritidae) under field-cage conditions. In *Proceedings of 6th International Fruit Fly Symposium*, 2002, pp. 99-104.

Muñoz-Barrios, R.; Cruz-López, L.; Rojas, J.C.; Hernández, E.; Liedo, P.; Gómez-Simuta, Y.; Malo, E. A. Influence of methoprene on pheromone emission and sexual maturation of *Anastrepha obliqua* (Diptera: Tephritidae) males. *J. Econ. Entomol.* **2016**, *109*, 637-643.

Quintero-Fong, L.; Toledo, J.; Ruiz, L.; Rendón, P.; Orozco-Dávila, D.; Cruz, L.; Liedo, P. Selection by mating competitiveness improves the performance of *Anastrepha ludens* males of the genetic sexing strain Tapachula-7. *Bull. Entomol. Res.* **2016,** *106*, 624-632.

Robacker, D.C.; Hart, W.G. (Z)-3 nonenol, (Z-Z)-3,6-nonadienol and (S,S)-(-)epianastrephin: male-produced pheromones of the Mexican fruit fly. *Entomol. Exp. Appl*. **1985**, *39*, 103-108.

Rocca, J.R.; Nation, J.L.; Strekowski, L.; Battiste, M.A. Comparison of volatiles emitted by male Caribbean and Mexican fruit flies. *J. Chem. Ecol.* **1992**, *18*, 223-244.

Stokes, J.B.; Uebel, E.C.; Warthen Jr, J.D.; Jacobson, M.; Flippen-Anderson, J.L.; Gilardi, R.; Spishakoff, L.M.; Wilzer, K.R. Isolation and identification of novel lactones from male Mexican fruit flies. *J. Agric. Food. Chem.* **1983**, *31*, 1162-1167.
